# Polo-like kinase 1 (Plk1) inhibition synergizes with taxanes in triple negative breast cancer

**DOI:** 10.1371/journal.pone.0224420

**Published:** 2019-11-21

**Authors:** Antonio Giordano, Yueying Liu, Kent Armeson, Yeonhee Park, Maya Ridinger, Mark Erlander, James Reuben, Carolyn Britten, Christiana Kappler, Elizabeth Yeh, Stephen Ethier

**Affiliations:** 1 Department of Medicine, Division of Hematology & Oncology, Medical University of South Carolina, Charleston, South Carolina, United States of America; 2 Department of Public Health Sciences, Hollings Cancer Center, Medical University of South Carolina, Charleston, South Carolina, United States of America; 3 Trovagene Oncology, San Diego, California, United States of America; 4 Department of Hematopathology, The University of Texas MD Anderson Cancer Center, Houston, Texas, United States of America; 5 Department of Pathology and Laboratory Medicine, Medical University of South Carolina, Charleston, South Carolina, United States of America; 6 Department of Pharmacology and Toxicology, Indiana University School of Medicine, Indianapolis, Indianapolis, United States of America; Columbia University, UNITED STATES

## Abstract

Within triple negative breast cancer, several molecular subtypes have been identified, underlying the heterogeneity of such an aggressive disease. The basal-like subtype is characterized by mutations in the *TP53* gene, and is associated with a low pathologic complete response rate following neoadjuvant chemotherapy. In a genome-scale short hairpin RNA (shRNA) screen of breast cancer cells, polo-like kinase 1 (Plk1) was a frequent and strong hit in the basal breast cancer cell lines indicating its importance for growth and survival of these breast cancer cells. Plk1 regulates progression of cells through the G2-M phase of the cell cycle. We assessed the activity of two ATP-competitive Plk1 inhibitors, GSK461364 and onvansertib, alone and with a taxane in a set of triple negative breast cancer cell lines and *in vivo*. GSK461364 showed synergism with docetaxel in SUM149 (Combination Index 0.70) and SUM159 (CI, 0.62). GSK461364 in combination with docetaxel decreased the clonogenic potential (interaction test for SUM149 and SUM159, p<0.001 and p = 0.01, respectively) and the tumorsphere formation of SUM149 and SUM159 (interaction test, p = 0.01 and p< 0.001). In the SUM159 xenograft model, onvansertib plus paclitaxel significantly decreased tumor volume compared to single agent paclitaxel (p<0.0001). Inhibition of Plk1 in combination with taxanes shows promising results in a subset of triple negative breast cancer intrinsically resistant to chemotherapy. Onvansertib showed significant tumor volume shrinkage when combined with paclitaxel *in vivo* and should be considered in clinical trials for the treatment of triple negative cancers.

## Introduction

Triple-negative breast cancer (TNBC), defined histologically as estrogen receptor negative, progesterone receptor negative and absence of *HER2/neu* amplification, represents 15–20% of all breast cancers and is characterized by an aggressive clinical course compared with other subtypes. Within TNBC, several molecular subtypes have been identified, underlying the heterogeneity of such an aggressive disease [1]. The heterogeneous nature of TNBC suggests that different TNBC subtypes may be associated with very different prognoses and, as described by Masuda et al, a wide range of pathologic complete response (pCR) rates were observed after neoadjuvant chemotherapy [2]. The basal-like 2 (BL2) subtype, identified for the first time by Lehmann and colleagues, is characterized by overexpression of epidermal growth factor receptor (EGFR), loss of PTEN, and mutations in the *TP53* gene. In a retrospective analysis conducted at the MD Anderson Cancer Center, patients with BL2 breast cancer had a 0% pCR rate following neoadjuvant chemotherapy. Thus, BL2 breast cancers are intrinsically resistant to chemotherapy and patients with this type of breast cancer have a poor overall survival rate. At the moment, a targeted therapeutic approach for the treatment of basal-like breast cancer patients does not exist, and patients receive standard chemotherapy with anthracycline, taxane and/or platinum compounds [3].

In a recent genome-scale shRNA (short hairpin RNA) screen of the SUM series of human breast cancer cell lines (www.sumlineknowledgebase.com), polo-like kinase 1 (Plk1) was a hit in several TNBC cell lines, indicating its importance for growth and survival of these breast cancer cells [4]. mRNA expression, reverse phase protein array and immunohistochemistry showed a higher expression of Plk1 in TNBC compared with other subtypes of breast cancer and healthy breast tissue [5, 6]. Plk1 regulates progression of cells through the G2 phase of the cell cycle by phosphorylating FOXM1, which then regulates the expression of cyclins and other genes necessary for cells to progress through the cell cycle [7–10]. Two papers provided clues to a mechanistic basis for Plk1 drug sensitivity. In the early pre-clinical development of Plk1 targeted drugs, it was observed that cancer cells with *TP53* mutations were more responsive and had lower IC_50_ than cell lines with wild type *TP53* [11]. These observations are consistent with the lack of checkpoint control and the genomic instability associated with *TP53* mutations, which increases the importance of Plk1 function for progression through G2 and M phases of the cell cycle. In addition, Tan et al [12] published data suggesting the importance of a signaling axis involving 3-phosphoinositide–dependent protein kinase-1 (*PDK1*), *Plk1*, and *MYC* in driving the expression of a set of genes associated with cancer stem cell (CSC) self-renewal. Thus, it is possible that blocking Plk1 function can, in addition to affecting the ability of cancer cells with unstable genomes to progress through mitosis, reduce the self-renewal capacity of cancer stem cells and in that way, increase the overall sensitivity of the cells to chemotherapy agents such as taxane and platinum derivatives.

A large number of anti-Plk1 agents have been developed and tested under various preclinical and clinical settings, and some of them are currently in clinical trials, with varying degrees of success [13–31]. One of the major problems associated with the currently available Plk1 ATP-competitive inhibitors is their low degree of selectivity against other kinases, and their toxicity that could be partly due to their interference with other kinases [13]. A new generation of anti-Plk1 agents that target the polo-box domain of Plks are currently being tested pre-clinically and have demonstrated improved specificity towards Plk1 [32]. GSK461364 (GlaxoSmithKline, Brentford, UK) is a potent, selective, and reversible ATP-competitive Plk1 inhibitor with at least a 390-fold greater selectivity for Plk1 than for Plk2 and Plk3 and a 1,000-fold greater selectivity than for a panel of 48 other kinases [33]. Onvansertib (Trovagene, San Diego, USA) is an orally available, highly selective Plk1 inhibitor currently under clinical investigation in solid tumors [34–40].

Based on the role of Plk1 in the basal subtype, and given the potential mechanisms for synergy between Plk1 inhibition and chemotherapy, we tested GSK461364 and onvansertib across a panel of TNBC cell lines alone and in combination with taxanes and cisplatin. Also, we tested the combination of onvansertib plus paclitaxel in a xenograft model of TNBC. Thus, we not only elucidated the relative sensitivity of a panel of breast cancer cell lines to Plk1 targeted drugs, but also attempted to determine if Plk1 inactivation sensitizes breast cancer cells to taxanes.

## Materials and methods

### Cell cultures

SUM cell lines were developed by Dr. Stephen P. Ethier and have been previously described [41]. We received these cell lines directly from Dr. Stephen P. Ethier’s laboratory, and the cells were maintained as previously described [42, 43]. DU4475 breast carcinoma cell line was from ATCC, maintained in standard media and used at low passages. MCF10A cells were a gift from Dr. Herb Soule at the Michigan Cancer Foundation [44]. Short-tandem repeat profiling of human cell lines is routinely performed in our laboratory (Genetica Cell Line Testing, Burlington, NC, USA).

#### SUM149 and SUM159 cell line characteristics

SUM149 cells were derived from an African American woman affected by locally advanced Inflammatory Breast Cancer, a type of breast cancer carrying poor prognosis and survival. SUM149 cells have a TP53p.M237I mutation, and as well has a mutation in BRCA1 that is not commonly associated with familial breast cancer. In addition, they have complete loss of expression of PTEN mRNA and protein without any changes in the coding sequence for the PTEN gene [45, 46]. SUM159 cells, derived by TNBC primary tumor, have become most widely used for their ability to generate a high fraction of breast cancer stem cells [47]. Expression profiling of SUM159 cells by Perou and others have put this cell line in the claudin-low subset of basal breast cancers, and it clusters very close to the MDA-MB-231 cell line, exhibiting mesenchymal features [48]. SUM159 cells exhibit a highly focal amplification of 8q24 that involves only the *MYC* gene resulting in high level amplification and over expression [45, 49, 50].

### Growth and clonogenic assays

For the growth assay, cells were plated in 6-well plates at a density of 100,000 cells per well. Cells were maintained in their normal growth medium for 24 hours before being treated in triplicate with a wide range of drug concentrations or DMSO. After a 3-day exposure to drug, cell number was determined by harvesting and counting nuclei with a Beckman Coulter Z1 Particle Counter. We performed this experiment with all cell lines using GSK461364, docetaxel and cisplatin. IC_50_ and IC_25_ for each drug were determined using CompuSyn software. To study synergy between drugs, cells were plated at the same density of the growth assay, and treated with the combination of GSK461364 at IC_25_ and chemotherapy, docetaxel or cisplatin, at IC_50_. For each drug combination and in each cell line, a total of six different concentrations were tested as follows: IC_25-GSK461364_ + IC_50-chemotherapy_, double concentration of same combination, 1/2, 1/4, 1/8 of concentrations, and control. CompuSyn software was utilized to calculate Combination Index (CI) for each combination and cell line [51–53].

For the colony-forming assay, cells were seeded in triplicate at clonal density in 6-well plates and treated with drugs at 24 hours after plating. At 72 hours, cells were washed and cultured in normal growth media for 7 to 14 days or until colony sizes reached over 50 cells. For staining, colonies were stained with 1 mL/well 0.2% crystal violet solution containing 3.7% paraformaldehyde for 15 minutes at room temperature and then de-stained with dH2O and air dried. Colony counts were determined using a GelCount^™^ colony counter (Oxford Optronix, Oxfordshire, United Kingdom). The average number of colonies per dish was used to determine the colony forming ability at each drug concentration as a measure of clonogenic potential of the cells.

### Tumorsphere assay

For the tumorsphere assays, cultured cells were plated at a density of 20,000 cells per well in ultra-low attachment 6 well plates (Corning^®^, USA) in serum-free medium. The serum-free medium consisted of Ham’s F-12 medium supplemented with 2% B27 (Thermo Fisher Scientific, USA), 25 μg/ml gentamicin (Gemini Bioproducts), 2.5 μg/ml fungizone (Thermo Fisher Scientific), 20 ng/ml EGF (Sigma-Aldrich, USA), 20 ng/ml bFGF (Sigma-Aldrich), 4 μg/ml heparin and 0.5 μg/ml hydrocortisone (Sigma-Aldrich, USA). During primary culture, cells were treated after 24 hours with dimethyl sulfoxide (DMSO, control), docetaxel IC_50_, GSK461364 IC_50_, or both in combination. After seven days, or when spheres diameter was > 60 um, formed spheres were counted using a Celigo Imaging Cytometer (Nexcelom, USA). The primary tumorspheres were harvested, dissociated with trypsin and collected. Five hundred dissociated cells were then cultured in ultra-low attachment 96 well plates (Costar^®^, USA) in serum-free medium for an additional seven days in the absence of drug. Secondary passage tumorspheres were then counted as before.

### Cell cycle, immunoblotting and DNA content analysis

For immunoblotting, apoptosis and DNA content analysis, cells were synchronized with double thymidine block. For synchronization, SUM149 and SUM159, were treated with 2 mmol/L thymidine, incubated for 24 hours, then washed with PBS before adding fresh media. After an 18-hour release, cells were again treated with thymidine for an additional 24 hours of incubation. Finally, cells were released for 2 hours in fresh media and treated with GSK461364, GSK461364 + docetaxel at the IC_50_ concentrations and DMSO. Cells were washed with PBS and lysed in RIPA buffer (Roche), Halt^™^ Protease Inhibitor Cocktail (100X) (Fisher) and PhosSTOP Phosphatase Inhibitor Cocktail Tablets (Roche). The lysates were run on a 4–12% gel (NuPage), transferred to a polyvinylidene difluoride membrane and sequentially probed with antibodies to Cyclin B1, CDK1, Phosphorus-histone H3 (Ser10), b-actin (Sigma), FoxM1, Cleaved Parp (Asp214), PLK1 (Cellsignaling), HRP-conjugated secondary antibodies (Bio Rad). Analysis was repeated at 8, 24, and 48 hours. For DNA content analysis, cells were synchronized and treated with drugs as same as immunoblotting. Cells were collected at 8, 24, and 48 hours and fixed in chilled 70% ethanol and stored at -20°C. Cells were washed twice with PBS, and then stained using 20 ug/ml propidium iodide, 0.2 mg/ml RNaseA, and 0.1% Triton X-100 in PBS. Data were acquired on BD Fortessa X-20 Analytic Flow Cytometer and analyzed with FlowJo software.

### In vivo experiment

All animal procedures were approved by the MUSC Institutional Animal Care and Use Committee (IACUC) under protocol number 2018–00674 (S1 File). Five million SUM159 cells were injected in the mammary fat pad of NOD-scid-IL2 receptor gamma null female mice (Jackson Laboratory). When tumors reached a volume higher than 40 mm^3^, 35 mice were randomized to receive onvansertib 120 mg/kg day 1–2 every week by oral gavage (P.O.), paclitaxel 10 mg/kg day 1 every week by intraperitoneal injection (I.P.), the combination of the two drugs at the same dose of single agents, or control vehicles. In each arm, P.O. and I.P. vehicle was given as per the control arm. Tumors were independently assessed by caliper by two researchers (A.G. and Y.L.) twice per week and treatments continued for 21 days.

### Statistical analysis

Experiments resulting in count data, such as colony forming assays and tumorsphere assays, were analyzed using poisson regression models. Interaction effects for drug combinations were included in all models, and linear combinations of the resulting model coefficients were used to estimate fold changes with 95% confidence intervals from the reference condition. Two-sided α = 0.05 was used for determining statistical significance. Regression analysis was performed using the R statistical software package [54]. The combined effects of different concentrations of GSK461364 with docetaxel and cisplatin were evaluated, and the CI values were determined using CompuSyn software [51–53]. Three biological repeats were performed for each in vitro experiment. For the animal experiments, a group sample size of eight per arm was determined to attain power of 80% to detect a standardized effect size of 2.003 based on a two-sample t-test with two-sided α = 0.05/6≈0.008. The primary endpoint for the animal experiments is tumor volume measured longitudinally. We fit a linear mixed effects (LME) regression model with (log-transformed) tumor volume as the response variable, and fixed effects for experimental condition, time, and their interaction. The logarithmic transformation of relative tumor volume is required to satisfy assumptions of approximate normality. Models will include mouse-specific random effects to account for the correlation among measures obtained from the same animal over time.

## Results

### Effect of GSK461364, docetaxel and cisplatin on proliferation of triple-negative breast cancer cells

**Fig 1:**
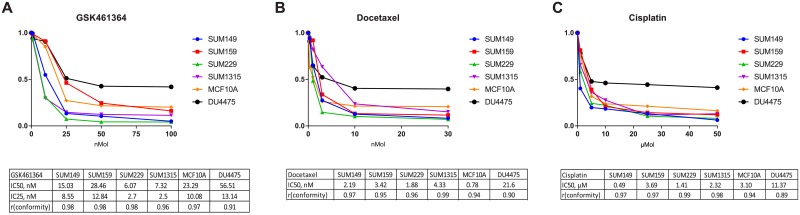
TNBC cell lines have diverse sensitivity to Plk1 inhibition and chemotherapy. TNBC cell lines: SUM149, SUM159, SUM229, SUM1315 and DU4475 cells, and the non-tumorigenic human breast epithelial cell line MCF10A. TNBC cell lines were treated for 72 hours in triplicates. Three replicate data were performed for each concentration and the best dose–response model (r conformity closest to 1) was selected and graphed. After a 72 hours exposure to drug, cell number was determined by harvesting and counting nuclei with a Beckman Coulter Z1 Particle Counter. IC_50_, IC_25_, and r conformity were determined using CompuSyn software. (A) Sensitivity to GSK461364, Plk1 inhibitor, in TNBC cell lines; IC_50_, IC_25_, and r conformity were reported for GSK461364. (B) TNBC cell lines sensitivity with respective IC_50_ and r conformity to docetaxel. (C) TNBC cell lines sensitivity with respective IC_50_ and r conformity to cisplatin.

The SUM lines showed a range of sensitivity to *Plk1* knock-down in an shRNA screening experiment previously completed (S1 Appendix Table 1) [4]. To study the effect of Plk1 inhibition in TNBC, we used the Plk1-selective inhibitor, GSK461364 [33]. We assessed the effects of GSK461364 on proliferation of TNBC SUM149, SUM159, SUM229, SUM1315 and DU4475 cells, and the non-tumorigenic human breast epithelial cell line MCF10A. DU4475 is known to be resistant to a wide range of chemotherapies. In a 72-hour growth inhibition assay, GSK461364 caused growth inhibition with IC_50_ values ranging from 6.1 nmol/L (SUM229) to 56.5 nmol/L (DU4475) (Fig 1A). Complete inhibition of cell proliferation was found in SUM229, SUM1315 and SUM149 cells treated with 25 nmol/L GSK461364. Single agent docetaxel and cisplatin IC_50s_ were obtained for the same cell lines. As expected, docetaxel was found to be highly active *in vitro* for all cell lines tested, with all cell lines having an IC_50_ < 22 nmol/L (Fig 1B). DU4475 was resistant to cisplatin with IC50 = 13.8 μmol/L (Fig 1C).

**Fig 2:**
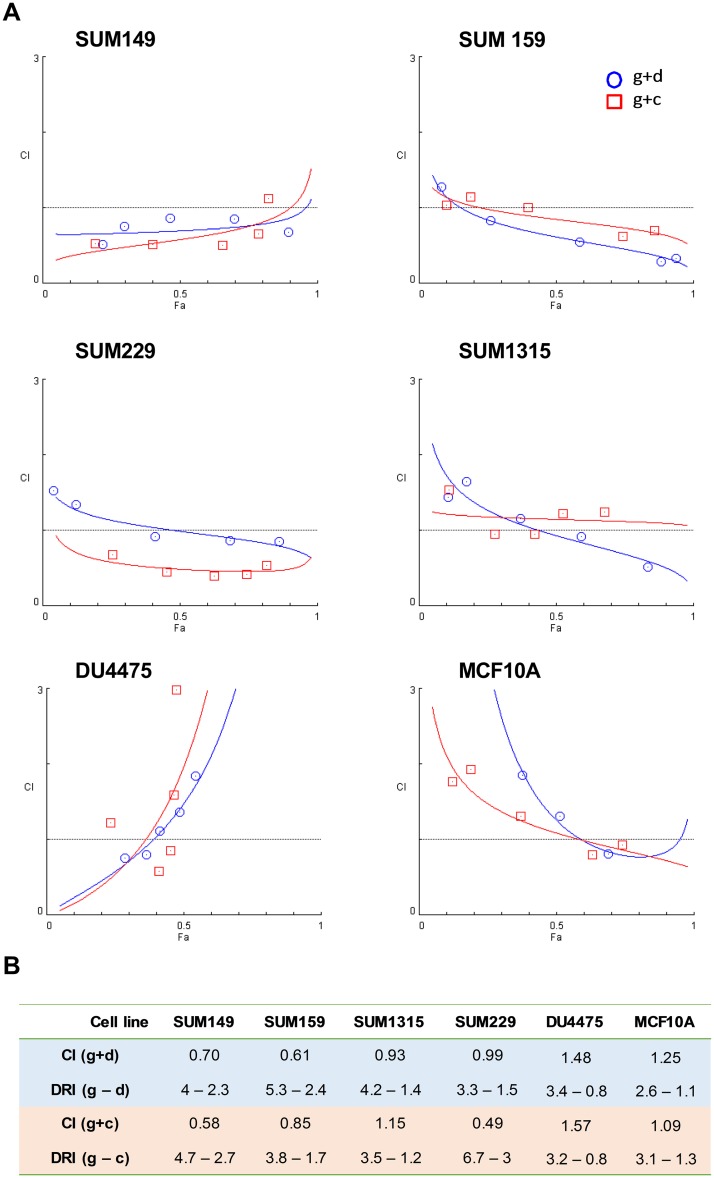
Plk1 inhibition synergizes with docetaxel and cisplatin in SUM149 and SUM159. (A), Combination index (CI) curves for the 6 cell lines treated with GSK461364 plus docetaxel or cisplatin. TNBC cell lines: SUM149, SUM159, SUM229, SUM1315 and DU4475 cells, and the non-tumorigenic human breast epithelial cell line MCF10A. TNBC cell lines were treated with the Plk1 inhibitor GSK461364 (g) at the IC_25_ dose plus each chemotherapy drug (d, docetaxel; c, cisplatin) at the IC_50_ concentration. (B), CI and DRI values for each combination of GSK461364 plus chemotherapy. CI and the dose-reduction index (DRI) values were determined using CompuSyn software. CI <1 indicates a synergism. DRI >1 indicates a favorable dose reduction.

Based on the role of Plk1 in the basal subtype, and given the potential mechanisms for synergy between Plk1 inhibition and chemotherapy targeting the G2-M phase, we combined GSK461364 at the IC_25_ dose with each chemotherapy drug at the IC_50_ concentration, and five other concentrations as described in methods. In our combination growth assay, GSK461364 showed synergism with docetaxel in SUM149 (CI, 0.7; DRI, 4) and SUM159 (CI, 0.61; DRI, 5.3), and with cisplatin in SUM149 (CI, 0.58; DRI, 4.7), SUM159 (CI, 0.85; DRI 3.8), and SUM229 (CI, 0.49; DRI, 3.5), respectively (Fig 2). Because of the synergistic combination of docetaxel and cisplatin with GSK461364 we have observed in SUM149 and SUM159 cells, we focused on these two cell lines in the next experiments. Based on Lehmann genomic classification of TNBC, SUM149 and SUM159 clustered in BL2 and mesenchymal-stem like subtypes, respectively [1]. The two latter subtypes of TNBC are known to have the lowest response to chemotherapy (0% and 23% pCR, respectively) [2].

### Onvansertib and paclitaxel synergizes in SUM149 and SUM159 cell lines

**Fig 3:**
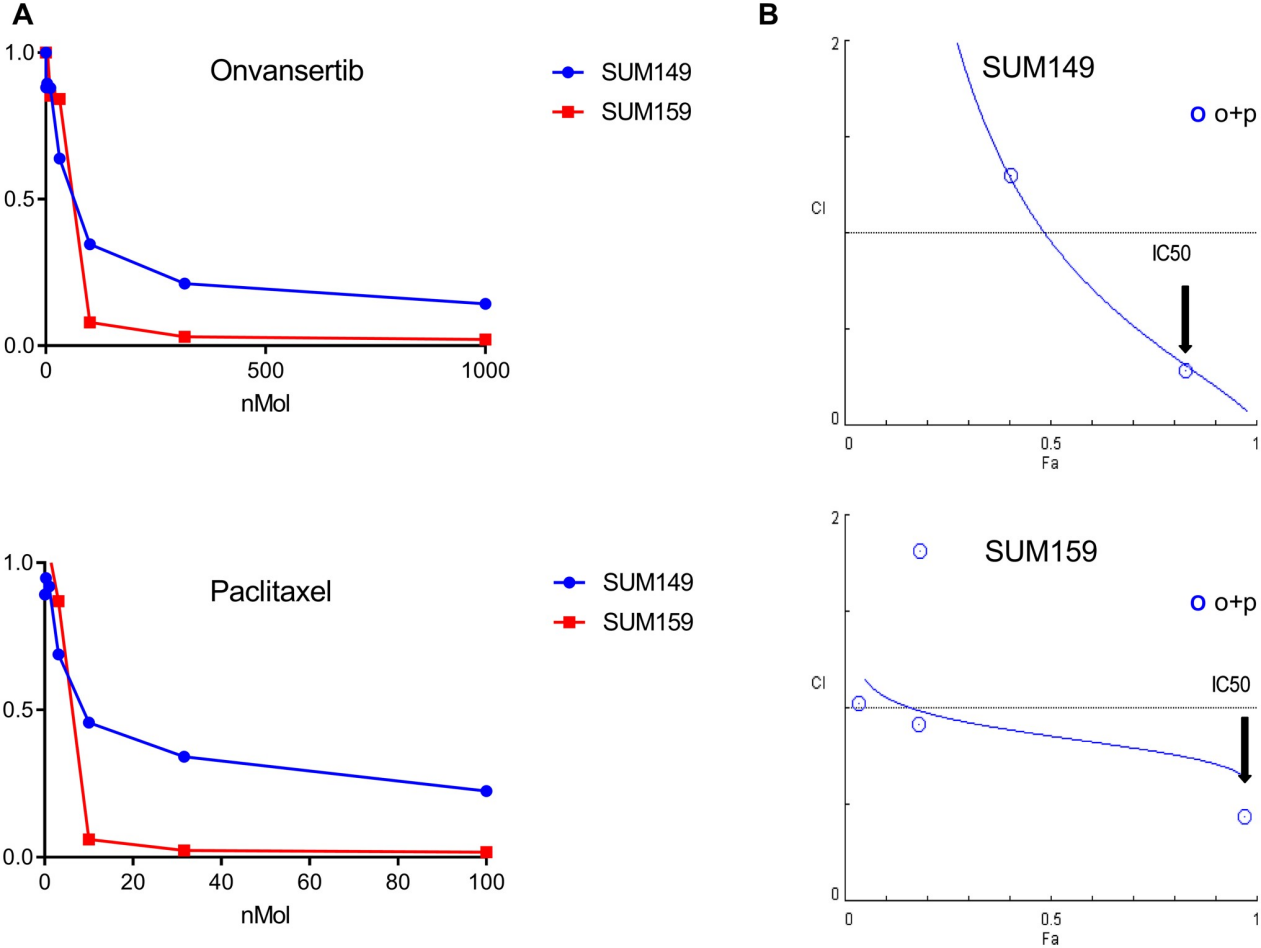
Onvansertib synergizes with paclitaxel in SUM149 and SUM159. SUM149 and SUM159 cells were treated for 72 hours in triplicates with onvansertib and paclitaxel (A). After a 72 hours exposure to drug, cell number was determined by harvesting and counting nuclei with a Beckman Coulter Z1 Particle Counter. IC_50_ was determined using CompuSyn software. SUM149 and SUM159 were treated with the Plk1 inhibitor onvansertib (o) at the IC_50_ dose plus paclitaxel (p) at the IC_50_ concentration (B). Combination Index (CI) values for each combination of onvansertib plus paclitaxel were determined using CompuSyn software. CI <1 indicates a synergism.

To confirm synergy between Plk1 inhibition and taxanes, we performed a cell proliferation assay with the Plk1 inhibitor onvansertib and paclitaxel in two cell lines, SUM149 and SUM159. In a 72-hour growth inhibition assay, onvansertib caused growth inhibition with IC_50_ of 48.5 nmol/L in SUM149 and 49.4 nmol/L in SUM159. Paclitaxel IC_50_ was 5.5 nmol/L and 4.1 nmol/L in SUM149 and SUM159, respectively (Fig 3A). In our combination growth assay, onvansertib showed synergism with paclitaxel in SUM149 (CI, 0.54) and SUM159 (CI, 0.54) (Fig 3B).

### Suppression of clonogenic potential by Plk1 inhibition in SUM149 and SUM159

**Fig 4:**
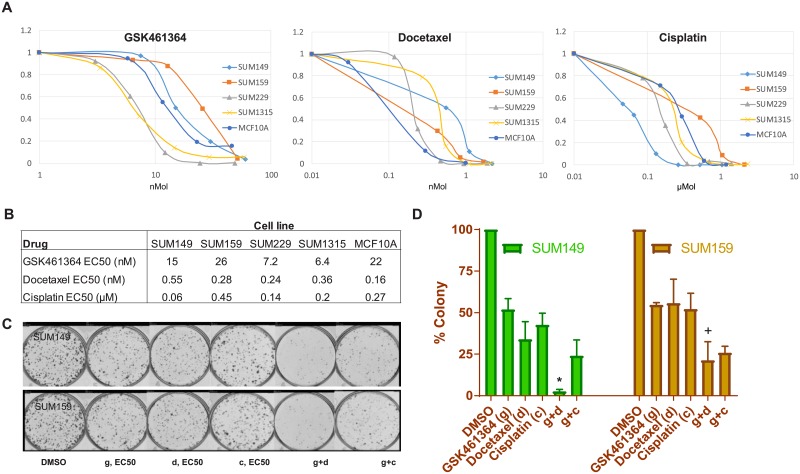
Plk1 inhibition suppresses the clonogenic potential of SUM149 and SUM159. (A) TNBC cell lines (SUM149, SUM159, SUM229, SUM1315 and DU4475 cells, and the non-tumorigenic human breast epithelial cell line MCF10A) were incubated with GSK461364, docetaxel, and cisplatin at the indicated concentrations for 24 hours; the medium was then changed to remove the drug, and colony formation was measured 7–14 days later. (B) EC_50_ was defined as 50% colony inhibition. Single agents EC_50_ were reported for each TNBC cell line and drug. According to the synergistic combination of docetaxel and cisplatin with GSK461364 we have observed in SUM149 and SUM159 cells, we focused on these two cell lines in the next experiments. (C), Colony forming assay photographs for single agents GSK461364, docetaxel, and cisplatin, with the respective combinations, in SUM149 and SUM159. (D) Histograms show colony formation rates, normalized to DMSO colony number for each cell line, for single drug and respective combinations; GSK461364 plus docetaxel interacted synergistically to decrease the clonogenic potential of SUM149 and SUM159 (p < 0.001 and p < 0.001, respectively; Interaction Test, p = 0.001* and p = 0.03^+^, respectively).

To determine if GSK461364 resulted in irreversible growth inhibition on SUM149 and SUM159 cells in addition to the cytostatic effects already demonstrated, clonogenic survival assays were performed. Cells that had been exposed to the drugs either singly or in combination were allowed to grow at clonal density for 7–14 days, which resulted in the formation of colonies of equivalent size as those seen with control cells grown for the same time. First, we determined the effect (EC_50_) of single agents GSK461364, docetaxel, and cisplatin on clonogenic capacity of the cell lines (Fig 4A), and then used data from those experiments to determine the effect of the drug combinations on clonogenic potential. GSK461364 plus docetaxel interacted synergistically to decrease the clonogenic potential of SUM149 and SUM159 (p < 0.001 and p < 0.001, respectively; Interaction Test, p = 0.001 and p = 0.03, respectively) (Fig 4B and 4C). There was a 13-fold difference in clonogenic potential with Plk1 inhibition compared to docetaxel alone in SUM149, and a 3-fold difference with Plk1 inhibition and docetaxel compared to docetaxel alone in SUM159. The results of the experiment performed in this way confirmed the results shown in Fig 2, indicating that the effect of GSK461364 plus docetaxel on the clonogenic potential of SUM149 and SUM159 cells is a true effect on cell survival and not the result of the cytostatic properties of the drug.

### When combined with docetaxel, GSK461364 reduced tumorsphere formation of SUM149 and SUM159

**Fig 5:**
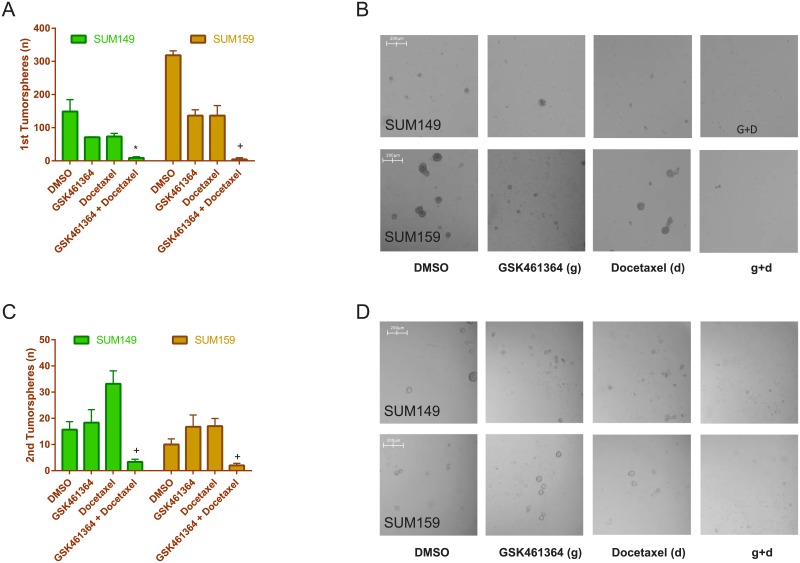
GSK461364 plus docetaxel reduce tumorsphere formation of SUM149 and SUM159. (A, histograms and B, pictures) For the primary tumorsphere assay, we analyzed the tumorsphere formation in presence or absence of docetaxel, GSK461364, and the combination at the IC_50_ concentrations identified in the proliferation assay; SUM149 and SUM159 cells were plated at a density of 20,000 cells per well in ultra-low attachment 6 well plates and after 24 hours treated with DMSO (control), docetaxel IC_50_, GSK461364 IC_50_, or both in combination. Formed spheres were counted using the Celigo Imaging Cytometer (Nexcelom, USA). GSK461364 plus docetaxel decreased primary tumorsphere formation of SUM149 and SUM159 (Interaction Test, p = 0.01* and p < 0.001^+^, respectively). (C, histograms and D, pictures) Dissociated tumorspheres (N = 500) were plated in ultra-low attachment 96 well plates (Costar^®^, USA) in serum-free medium for an additional seven days in the absence of drug. Secondary passage tumorspheres were then counted as before. GSK461364 plus docetaxel decreased primary tumorsphere formation of SUM149 and SUM159 (Interaction Test, p < 0.001^+^ and p < 0.001^+^, respectively). Poisson regression model with drug interaction was used to assess statistical differences.

There is evidence that breast cancer cells with a stem cell-like phenotype are metastasis-forming cells in breast cancer [55]. Both SUM149 and SUM159 cells are able to generate tumorspheres *in vitro* [56]. To explore the synergistic effect of chemotherapy plus Plk1 inhibition on the stem cell activity and self-renewal potential of these cell lines, we analyzed the tumorsphere formation in presence or absence of docetaxel, GSK461364, and the combination. At the IC_50_ concentration identified in the proliferation assay, docetaxel plus GSK461364 significantly reduced primary tumorsphere formation in both SUM149 and SUM159 (0.06 and 0.02-fold decrease of control levels, respectively; interaction test, p = 0.01 and p< 0.001, respectively, as shown in S1 Appendix Table 2) (Fig 5A). During the secondary assay, cells treated with single agent docetaxel demonstrated an increased proportion of tumorspheres, to a 2.12-fold of control levels in SUM149 cells and to a 1.7-fold of control levels in SUM159 cells. After combination of docetaxel plus GSK461364, the number of tumorspheres decreased to a 0.21-fold in SUM149 and a 0.2-fold in SUM159 of control levels (Fig 5B) (Table 1). In the analysis of cancer stem cells marker, we found that the combination GSK461364 plus docetaxel decreased the number of CD44^+^/CD24^-/dim^ in SUM149 (from 26% in DMSO to 18% after treatment, S1 Appendix Fig 1A) and SUM159 (from 89% in DMSO to 77% after treatment, S1 Appendix Fig 1B) compared to control (DMSO). We demonstrated that Plk1 inhibition plus docetaxel can reduce CSC formation of SUM149 and SUM159 cells and that Plk1 inhibition can sensitize otherwise resistant TNBC cells to chemotherapy.

**Table 1:** Statistical analysis for secondary tumorsphere formation assay.

Condition	Fold Change	95% C.I.	p-value
**SUM149**			
DSMO	1 (reference)	NA	
GSK461364	1.17	0.89–1.55	0.26
Docetaxel	2.12	1.65–2.71	<0.001
GSK461364+Docetaxel	0.21*	0.13–0.35	<0.001
*Interaction p-value < 0.001			
**SUM159**			
DSMO	1 (reference)	NA	
GSK461364	1.67	1.13–2.49	0.01
Docetaxel	1.70	1.14–2.52	0.01
GSK461364+Docetaxel	0.20*	0.09–0.43	<0.001

*Interaction p-value < 0.001

C.I., confidence intervals; NA, not applicable.

### Effect of GSK461364 on cell cycle, apoptosis and DNA content in SUM149 and SUM159 cells

**Fig 6:**
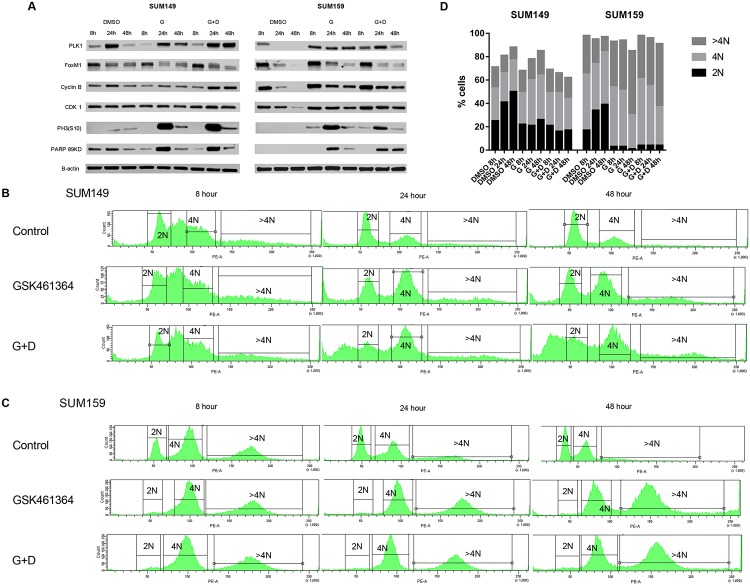
Cell cycle immunoblotting, apoptosis and DNA content analysis. For immunoblotting, apoptosis and DNA content analysis, cells were synchronized with double thymidine block. Cells were released for 2 hours in fresh media and treated with GSK461364 (G), GSK461364 + docetaxel (G+D) at the IC_50_ concentrations, and DMSO (control). (A) Immunoblotting of Plk1, FoxM1, Cyclin B1, CDK1, Phosphorus-histone H3 (Ser10), Cleaved PARP (89KDa), and β-actin were repeated at 8, 24, and 48 hours. For DNA content analysis, SUM149 (B) and SUM159 (C) cells were collected at 8, 24, and 48 hours and stained with propidium iodide. Data were acquired on BD Fortessa X-20 Analytic Flow Cytometer and analyzed with FlowJo. DNA copy content data showed in B and C were summarize in the histograms in D.

The effect of Plk1 inhibition on the cell cycle has been well characterized, and it typically leads to prometaphase arrest. Consistent with previous results [57], the biological effects of Plk1 inhibitor GSK461364A are highly dose, time, and cell line dependent. To investigate the effect of GSK461364 plus docetaxel on cell cycle at different time points, SUM149 and SUM159 were synchronized by double thymidine block and after 2 hours release, treated with control (DMSO), drugs alone or in combination. Protein lysates at different time points (8, 24 and 48 hours) after drug treatment were examined for expression levels of the cell cycle markers PLK1, FOXM1, Cyclin B1, CDK1, and phosphorylated histone H3 (pH3), as well as apoptosis by identification of cleaved PARP (89 KDa) (Fig 6A). GSK461364 alone, and with docetaxel, induced increased expression of PLK1. This phenomena could be consequent to attempted override of drug-induced PLK1 inhibition through a positive feedback mechanism of the protein [35]. GSK461364 induced mitotic arrest (Cyclin B1 and PLK1 accumulation followed by pH3 accumulation) earlier in SUM159 compared to SUM149. This difference could be explained by the fast doubling time and earlier mitosis entry by SUM159 compared to SUM149. Treatment with single PLK1 inhibitor induced apoptosis after 24 hours in both cell lines (Fig 6A). In the DNA content analysis with propidium iodide, the percentage of aneuploid SUM149 (Fig 6B) and SUM159 cells (Fig 6C) increased over time (24–48 hours) after treatment with GSK461364, and even more considerably after combination of GDK461364 with docetaxel. Indeed, greater aberrant mitotic exit and apoptosis was observed when SUM149 and SUM159 cells were treated with the combination of Plk1 inhibitor plus docetaxel (Fig 6D).

### Onvansertib plus paclitaxel leads to decreased breast cancer tumor size in vivo

**Fig 7:**
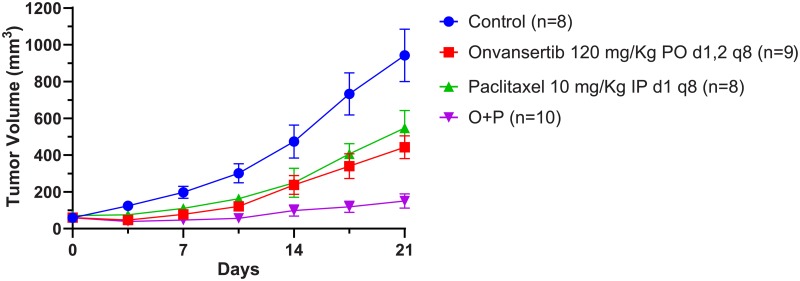
In vivo efficacy of onvansertib alone or in combination with paclitaxel against SUM159 xenografts. SUM159 cells were implanted in the mammary fat pad of NOD-scid-IL2 receptor gamma null female mice, and treatments began 14–21 days later when tumors were well established (tumor volume ≥ 40 mm3). Onvansertib was given by oral gavage (PO) on two consecutive days every week; paclitaxel was given intraperitoneally (IP) once per week; controls received PO vehicle on two consecutive days every week and IP vehicle once per week. Tumor volume was assessed twice per week and mice treated for 3 weeks. Mean of tumor volume with standard errors of the mean were plotted.

We examined the effect of PLK1 inhibition via treatment with onvansertib, an oral available PLK1 inhibitor, alone or in combination with paclitaxel on mouse xenograft models of the mesenchymal breast cancer SUM159. We choose paclitaxel for its better myelotoxicity profile in a weekly schedule in human [58]. A total of 35 mice were randomized to receive P.O. vehicle plus I.P. vehicle (N = 8), P.O. onvansertib 120 mg/Kg on day 1 and 2 every week plus I.P. vehicle (N = 9), I.P. paclitaxel 10 mg/Kg on day 1 every week plus P.O. vehicle (N = 8), P.O. onvansertib 120 mg/Kg on day 1 and 2 every week plus I.P. paclitaxel 10 mg/Kg on day 1 every week (N = 10) (Fig 7 and S1 Appendix Fig 2). The LME regression model provides all fixed effects for experimental condition (onvansertib, paclitaxel, onvansertib plus paclitaxel and control), time (3, 7, 10, 14, 18 and 21 days), and their interaction are significant (p-value < 0.0001 in all effects) (S1 Appendix Table 3). Onvansertib and paclitaxel demonstrated similar tumor growth inhibition when compared to controls (difference = -0.406 and -0.337 with p = 0.262 and 0.340, respectively, at 21 days). The combination onvansertib plus paclitaxel was significantly superior to single agent treatment with difference of -1.346 compared to onvansertib alone and -1.414 compared to paclitaxel alone (p<0.0001 and p<0.0001, respectively) (S1 Appendix Table 3).

## Discussion

In our study we showed that Plk1 inhibition synergizes with taxane and cisplatin in SUM149 (basal-like subtype), and in SUM159 cells (mesenchymal subtype), two subtypes of TNBC known to have poor response to conventional chemotherapy [59]. The results of the growth assay showed a favorable dose reduction of two Plk1 inhibitors, GSK461364 and onvansertib, when combined with a taxane. Also, we found that GSK461364 plus docetaxel was able to significantly reduce the clonogenic potential and the stem cell fraction of SUM149 and SUM159 cells. SUM149 and SUM159 are relatively sensitive to cisplatin and synergy with GSK461364 was not observed in our colony forming assay. The combination should be explored in additional cell lines more resistant to cisplatin. Finally, onvansertib, an oral highly selective Plk1 inhibitor, demonstrated in vivo activity in a xenograft model of TNBC, especially when in combination with paclitaxel.

Based on the fact that Plk1 is required for mitotic entry during recovery from G2 arrest induced by DNA damage [60], and based on our preliminary data in which *Plk1* is a functional key gene in the basal-like cell line SUM149, we demonstrated that Plk1 inhibition synergized with chemotherapies such as taxane, specifically inhibiting the G2-M transition, inducing aberrant mitotic exit, and apoptosis and the elimination of stem cell-like resistant tumor clones. Taxanes bind β-tubulin and induce mitotic arrest and apoptosis in actively dividing cells by inhibiting microtubule depolymerization [61]. As described in detail previously [13], *Plk1* contributes to paclitaxel resistance via its ability to regulate microtubule dynamics and microtubule–kinetochore attachment. Overall, approximately 20%–40% of TNBC patients achieve a pCR after standard anthracycline plus cyclophosphamide and taxane-based neoadjuvant chemotherapy [62]. Basal-like and mesenchymal subtypes have been shown to have lower rates of pCR (0–23%) compared to other subtypes of TNBC [2]. Based on the tumorsphere control of GSK461364 plus docetaxel, we hypothesize that combination of a Plk1-targeted drug plus a taxane will increase the pCR rate and thus survival of TNBC patients.

The sensitivity to Plk1 inhibition toward p53-deficient tumors compared with that of wild type p53 tumors could potentially offer an opportunity to treat breast tumors that are refractory to standard chemotherapy such as basal-like TNBC. Several papers showed that Plk1 interacts with the tumor suppressor p53 and that Plk1 is a direct transcriptional target of p53 [63–65]. In agreement with our data, Tan et al. described in detail previously [12] that PLK1 inhibition in highly invasive breast cancer MDA-MB-231 cells resulted in depletion of CSC-like CD44^+^/CD24^−/low^ populations and accordingly significantly reduced tumorsphere formation. SUM149 and SUM159, like most TNBC cell lines [66], have TP53 mutations and were demonstrated to be sensitive to Plk1 inhibition in combination with taxanes. Supported by consistent results published in the literature [11, 63–65, 67], preclinical results warrant testing *TP53* status as hypothetical biomarker of response to Plk1 inhibitors in the clinical setting. Because of the off-target toxicity encountered in the phase I trial of GSK461364 [33], we have decided to not utilize this drug *in vivo*. After we confirmed synergy in vitro, we combined onvansertib with paclitaxel for the treatment of xenograft models with SUM159 cells, known to be particularly aggressive and resistant to taxanes [68]. Onvansertib was found to have similar activity than paclitaxel in vivo, however in combination, onvansertib plus paclitaxel significantly reduced tumor volume compared to single agents.

To date, the majority of clinical trials with Plk1 inhibitors in solid tumors have been discontinued because of the serious side effects and drug limiting toxicity experienced with these drugs as monotherapy. GSK461364 has shown off-target effects and its clinical development has been placed on hold, specifically, due to the high incidence (20%) of venous thromboembolism observed in the phase I trial. Further clinical evaluation of GSK461364 should involve administration of prophylactic anticoagulation [33]. Volasertib (Boehringer Ingelheim, Ingelheim, Germany), an ATP-competitive kinase inhibitor with higher potency and selectivity, is the Plk1 inhibitor in the most advanced phase in clinical trials. Volasertib synergized with microtubule-destabilizing drugs, such as taxanes and vinorelbine, in preclinical rhabdomyosarcoma and Ewing sarcoma models [39, 69]. However, based on several phase I-II clinical trials [16–24], volasertib has been discontinued from clinical experimentation in solid tumors due to hematologic toxicity and low response rate when utilized as single agent. Onvansertib is the only Plk1 inhibitor currently under investigation in clinical trials for solid tumors, and its safety profile was characterized in a Phase I dose escalation study in advanced and metastatic solid tumors [40]. Onvansertib is being evaluated in combination with abiraterone and prednisone in adult patients with metastatic castration-resistant prostate cancer (NCT03414034), and in combination with FOLFIRI and bevacizumab in metastatic colorectal cancer patients with a Kras mutation (NCT03829410). Our preclinical results and the safety profile from the Phase I trial by Weiss et al. support the investigation of Plk1 inhibition with onvansertib in combination with taxane for the treatment of TNBC patients and TP53 mutations as biomarker of response.

## Conclusions

Based on the dramatic synergy we found at a favorable dose reduction and the ability to reduce CSCs of TNBC cells, the combination of a Plk1 inhibitor plus a taxane should be further investigated in a clinical trial for TNBC patients. Taken together, our results showed a substantial synergy of Plk1 inhibition and chemotherapy in basal-like and mesenchymal TNBC and warrant development of a clinical trial with onvansertib in combination with paclitaxel in a subset of breast cancer that currently carries a poor prognosis, and for which no targeted therapies exist.

## Supporting information

S1 Appendix Additional tables (Tables 1–3) and figures (Figs 1 and 2). (DOCX)

S1 File MUSC Institutional Animal Care and Use Committee (IACUC) protocol 2018–00674. (PDF)
