# Supplementary material for: Polo-like kinase 1 (Plk1) inhibition synergizes with taxanes in triple negative breast cancer
Source: PLoS One. 2019 Nov 21;14(11):e0224420. doi: 10.1371/journal.pone.0224420 (PMC6872222; doi:10.1371/journal.pone.0224420)
Supplement: S1 Appendix — (DOCX) [file pone.0224420.s001.docx]

**Supporting Information**

**S1 Appendix**

**Table 1. Quant-log hit score ranking of Plk1 in different SUM cell lines.**

| **Cell line** | **Plk1 Hit Score Ranking (16,000 genes)** |
| --- | --- |
| SUM149 | 334 |
| SUM229 | 2332 |
| SUM102 | 5556 |
| SUM159 | 2729 |
| SUM1315 | 9209 |
| SUM225 | 1011 |
| SUM190 | 980 |
| SUM44 | 2449 |
| SUM52 | 279 |
| SUM185 | 2274 |

Methods for shRNA analysis can be found in the paper by Kappler et al. (ref #4)

**Table 2. Statistical analysis for primary tumorsphere formation assay**

| **Condition** | **Fold Change** | **95% C.I.** | **p-value** |
| --- | --- | --- | --- |
| **SUM149** |  |  |  |
| DSMO | 1 (reference) | NA |  |
| GSK461364 | 0.44 | 0.36 - 0.54 | <0.001 |
| Docetaxel | 0.28 | 0.23 - 0.36 | <0.001 |
| GSK461364+Docetaxel* | 0.06 | 0.04 - 0.10 | <0.001 |
|  | | | |
| **SUM159** |  |  |  |
| DSMO | 1 (reference) | NA |  |
| GSK461364 | 0.31 | 0.26 - 0.36 | <0.001 |
| Docetaxel | 0.58 | 0.51 - 0.66 | <0.001 |
| GSK461364+Docetaxel^¥^ | 0.02 | 0.01 - 0.04 | <0.001 |

*P-value for interaction = 0.01

^¥^P-value for interaction <0.001

**Table 3. Statistical analysis for in vivo experiments**

General linear hypothesis tests were performed to see the differences in slopes between conditions using linear combinations of the fitted regression coefficients.

| comparison | estimate | standard error | t value | p value |
| --- | --- | --- | --- | --- |
| Onvansertib vs. Placebo | -0.019 | 0.017 | -1.123 | 0.263 |
| Paclitaxel vs. Placebo | -0.016 | 0.017 | -0.955 | 0.341 |
| Onvansertib+ Paclitaxel vs. Placebo | -0.083 | 0.016 | -5.084 | **<0.0001** |
| Onvansertib+ Paclitaxel vs. Onvansertib | -0/064 | 0.016 | -3.971 | **<0.0001** |
| Onvansertib+ Paclitaxel vs. Paclitaxel | -0.067 | 0.016 | -4.283 | **<0.0001** |

Comparisons between conditions will be performed using model-based linear contrasts at each time point.

| Time points | comparison | estimate | standard error | t value | p value |
| --- | --- | --- | --- | --- | --- |
| 3 days | Onvansertib vs. Placebo | -0.068 | 0.060 | -1.123 | 0.262 |
|  | Paclitaxel vs. Placebo | -0.056 | 0.059 | -0.955 | 0.340 |
|  | Onvansertib+ Paclitaxel vs. Placebo | -0.292 | 0.057 | -5.084 | **<0.0001** |
|  | Onvansertib+ Paclitaxel vs. Onvansertib | -0.224 | 0.056 | -3.971 | **<0.0001** |
|  | Onvansertib+ Paclitaxel vs. Paclitaxel | -0.236 | 0.055 | -4.283 | **<0.0001** |
| 7 days | Onvansertib vs. Placebo | -0.135 | 0.120 | -1.123 | 0.262 |
|  | Paclitaxel vs. Placebo | -0.112 | 0.118 | -0.955 | 0.340 |
|  | Onvansertib+ Paclitaxel vs. Placebo | -0.584 | 0.115 | -5.084 | **<0.0001** |
|  | Onvansertib+ Paclitaxel vs. Onvansertib | -0.449 | 0.113 | -3.971 | **<0.0001** |
|  | Onvansertib+ Paclitaxel vs. Paclitaxel | -0.471 | 0.110 | -4.283 | **<0.0001** |
| 10 days | Onvansertib vs. Placebo | -0.193 | 0.172 | -1.123 | 0.262 |
|  | Paclitaxel vs. Placebo | -0.161 | 0.168 | -0.955 | 0.340 |
|  | Onvansertib+ Paclitaxel vs. Placebo | -0.834 | 0.164 | -5.084 | **<0.0001** |
|  | Onvansertib+ Paclitaxel vs. Onvansertib | -0.641 | 0.161 | -3.971 | **<0.0001** |
|  | Onvansertib+ Paclitaxel vs. Paclitaxel | -0.673 | 0.157 | -4.283 | **<0.0001** |
| 14 days | Onvansertib vs. Placebo | -0.270 | 0.241 | -1.123 | 0.262 |
|  | Paclitaxel vs. Placebo | -0.225 | 0.235 | -0.955 | 0.340 |
|  | Onvansertib+ Paclitaxel vs. Placebo | -1.168 | 0.230 | -5.084 | **<0.0001** |
|  | Onvansertib+ Paclitaxel vs. Onvansertib | -0.897 | 0.226 | -3.971 | **<0.0001** |
|  | Onvansertib+ Paclitaxel vs. Paclitaxel | -0.943 | 0.220 | -4.283 | **<0.0001** |
| 17 days | Onvansertib vs. Placebo | -0.348 | 0.310 | -1.123 | 0.262 |
|  | Paclitaxel vs. Placebo | -0.289 | 0.303 | -0.955 | 0.340 |
|  | Onvansertib+ Paclitaxel vs. Placebo | -1.501 | 0.295 | -5.084 | **<0.0001** |
|  | Onvansertib+ Paclitaxel vs. Onvansertib | -1.153 | 0.290 | -3.971 | **<0.0001** |
|  | Onvansertib+ Paclitaxel vs. Paclitaxel | -1.212 | 0.283 | -4.283 | **<0.0001** |
| 21 days | Onvansertib vs. Placebo | -0.406 | 0.361 | -1.123 | 0.262 |
|  | Paclitaxel vs. Placebo | -0.337 | 0.353 | -0.955 | 0.340 |
|  | Onvansertib+ Paclitaxel vs. Placebo | -1.751 | 0.344 | -5.084 | **<0.0001** |
|  | Onvansertib+ Paclitaxel vs. Onvansertib | -1.346 | 0.339 | -3.971 | **<0.0001** |
|  | Onvansertib+ Paclitaxel vs. Paclitaxel | -1.414 | 0.330 | -4.283 | **<0.0001** |

**Figure 1. Cancer Stem Cell Markers analysis of SUM149 (A) and SUM159 (B).**


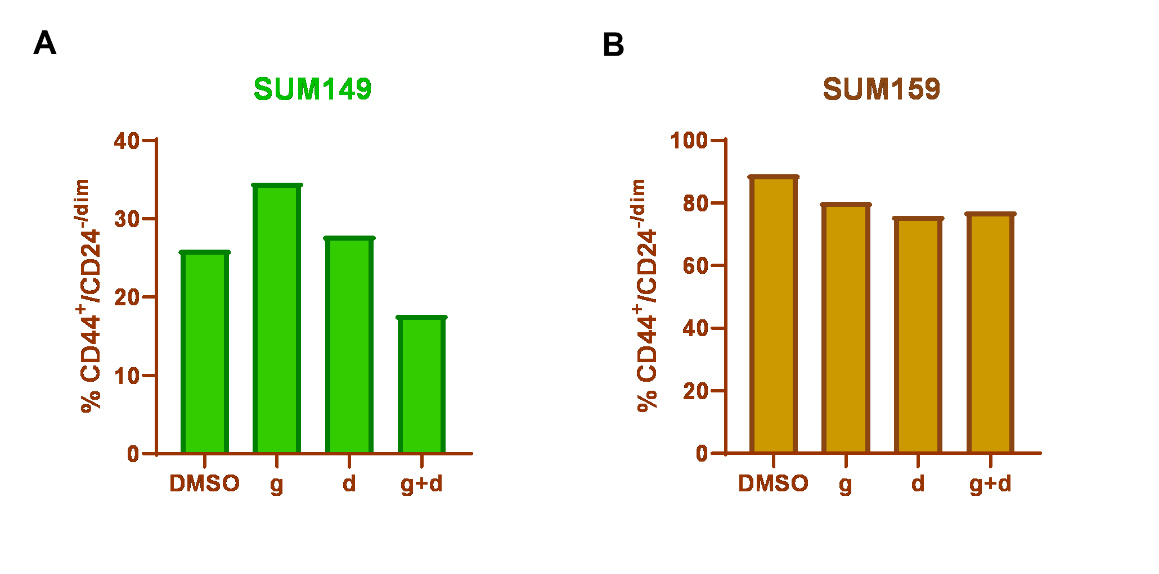


For cancer stem cell markers, we analyzed the percentage of CD44-positive and CD24-negative or low cells after treatment with GSK461364 (g), docetaxel (d), and the combination (g+d) at the IC50 concentrations identified in the proliferation assay; SUM149 and SUM159 cells were plated at a density of 20,000 cells per well in ultra-low attachment 6 well plates and after 24 hours treated with DMSO (control), docetaxel IC50, GSK461364 IC50, or both in combination. Data were acquired on BD Fortessa X-20 Analytic Flow Cytometer and analyzed with FlowJo.

**Figure 2. Tumor Volume Curves for each arm of the in vivo experiments.**


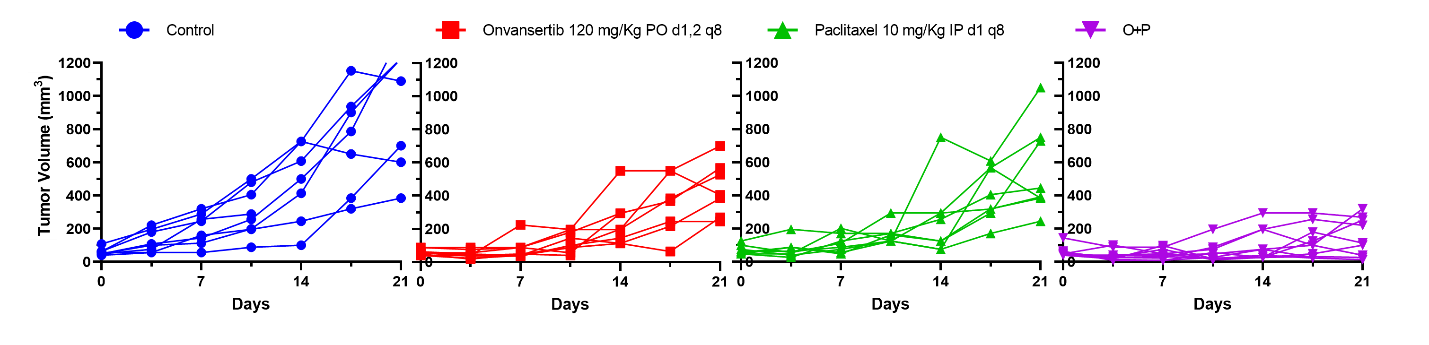


SUM159 cells were implanted in the mammary fat pad of NOD-scid-IL2 receptor gamma null female mice, and treatments began 14-21 days later when tumors were well established (tumor volume ≥ 40 mm3). Onvansertib was given by oral gavage (PO) on two consecutive days every week; paclitaxel was given intraperitoneally (IP) once per week; controls received PO vehicle on two consecutive days every week and IP vehicle once per week. Tumor volume was assessed twice per week and mice treated for 3 weeks. Longitudinal measurements of tumor volume per each mouse were plotted for each arm.
