# Supplementary material for: Polo-like kinase 1 (Plk1) inhibition synergizes with taxanes in triple negative breast cancer
Source: PLoS One. 2019 Nov 21;14(11):e0224420. doi: 10.1371/journal.pone.0224420 (PMC6872222; doi:10.1371/journal.pone.0224420)
Supplement: S1 File — (PDF) [file pone.0224420.s002.pdf]

Personnel Information 1

Species 5

Special Considerations 7

Funding 10

Rationale 11

Procedures 12

Alternative Search 22

Timing & Endpoints 24

Husbandry 25

Disposition of Animals 25

Attachments 26

Guidelines 27

Protocol Title: Polo-like kinase 1 (Plk1) inhibition for the treatment of breast cancer
Protocol Type: IACUC
Approval Period: 02/26/2019-02/25/2022
Important Note: This Print View may not reflect all comments and contingencies for approval. Please check the comments section of the online protocol.

* * * Personnel Information * * *

NOTE: PROTOCOL QUESTIONS AND INSTRUCTIONS ARE IN BOLD BLUE TEXT.
INVESTIGATOR RESPONSES ARE IN BLACK TEXT.
Click Next to advance to every section of the protocol. Every time you click Next, your changes will be saved.

PRINCIPAL INVESTIGATOR

The Principal Investigator can view, edit, and submit protocol.

Principal Investigator

Name*	Degree(s)
Antonio Giordano	MD PhD
**Email***	**Cell/Home Phone***
giordana@musc.edu	8328589804
**Office Location (Bldg/Room)***	**Office Phone***
BE429	843-792-4271
**Lab Location (Bldg/Room)**	**Lab Phone**
BE429	62385
**Department***	**Urgent Notification Preference**
Medicine	PI is primary contact for all urgent issues.

Describe general experience/training related to the proposed animal model(s) and procedure(s).*

PI has direct experinece of animal, both mouse and rat, treatment and procedures as graduate student.
Miami Citi training completed Working with the IACUC 05/29/2018.

Will the PI personally perform any animal work described in this protocol?* Y

Training Details

Course	CourseCompletionDate	CourseExpirationDate	UserID
Working with Mice in Research Settings	12/3/2018 1:28:52 PM		900130118
Reducing Pain and Distress in Laboratory Mice and Rats	12/3/2018 1:42:21 PM		900130118
Working with the IACUC	5/29/2018 1:57:33 PM		900130118
Group 1. Biomedical Investigators and Key Personnel	7/7/2017 3:20:13 PM	7/6/2020 3:20:13 PM	900130118
Good Clinical Practice and ICH	8/25/2016 11:37:34 AM	8/25/2019 11:37:34 AM	900130118
Group 1. Biomedical Investigators and Key Personnel	7/4/2014 11:21:27 AM	7/3/2017 11:21:27 AM	900130118

Protocol Title: Polo-like kinase 1 (Plk1) inhibition for the treatment of breast cancer

CO-INVESTIGATOR(S)

The Co-Investigator(s) can create, view, edit, and submit protocol and animal orders. Click the Add button to add multiple Co-Investigators. Leave blank if you do not have a Co-Investigator.

Co-Investigator

Name	Affiliation	Will this person have direct contact with a live animal or assume direct responsibility for some aspect of the care and use of live animals under this protocol?
Elizabeth Yeh	Faculty	Y

Co-Investigator

Name*
Elizabeth Yeh

Degree(s)
PhD

Email*
yeh@musc.edu

Phone*
843-876-2301

Affiliation*
Faculty

Notification Preference
Copy Co-I on all correspondence related to protocol.

Will this person have direct contact with a live animal or assume direct responsibility for some aspect of the care and use of live animals under this protocol?*
Y

Describe general experience/training related to animal research. Species- and procedure-specific experience will be addressed in the "Procedures" section of the application.
Dr. Yeh has worked with mouse models of cancer for ~15 years

Training Details			
Course	CourseCompletionDate	CourseExpirationDate	UserID
Group 1. Biomedical Investigators and Key Personnel	4/23/2018 9:27:02 AM	4/22/2021 9:27:02 AM	900130266
Principal Investigator or Lab Safety Representative	10/9/2017 4:02:26 PM		900130266
Animals in Biosafety	10/9/2017 10:28:53 AM		900130266
Personnel on an IBC Registration	10/9/2017 3:40:16 PM		900130266
On-Campus Assessment: Surgeon for Rodent Survival Surgery	5/5/2016 4:10:35 PM		900130266
Rodent Survival Surgery	10/27/2015 3:33:33 PM		900130266
Working with the IACUC	10/15/2013 4:27:00 PM		900130266
Reducing Pain and Distress in Laboratory Mice and Rats	10/15/2013 8:50:36 AM		900130266
Working with Mice in Research	10/15/2013 1:29:14 PM		900130266
Working with the IACUC	10/15/2013 4:27:00 PM		900130266
Working with Mice in Research Settings	10/15/2013 1:29:14 PM		900130266

LAB MANAGER

The Lab Manager can create, view, edit, and submit protocol and animal orders. Click the Add button to add multiple Lab Managers. Leave blank if you do not have a Lab Manager.

Lab Manager

Name	Affiliation	Will this person have direct contact with a live animal or assume direct responsibility for some aspect of the care and use of live animals under this protocol?
Yueying Liu	Staff	Y

Lab Manager

Name*
Yueying Liu

Degree(s)

Email*
liu@musc.edu

Phone*
8438762385

Title
Research Specialist II

Affiliation*
Staff

Protocol Title: Polo-like kinase 1 (Plk1) inhibition for the treatment of breast cancer

Will this person have direct contact with a live animal or assume direct responsibility for some aspect of the care and use of live animals under this protocol?* Y

Describe general experience/training related to animal research. Species- and procedure-specific experience will be addressed in the "Procedures" section of the application.

Yueying Liu is Dr. Giordano's technician and will handle all the animal handling in the Giordano Lab. She has 10+ years experience handling mice in a research setting.

Training Details

Course	CourseCompletionDate	CourseExpirationDate	UserID
Reducing Pain and Distress in Laboratory Mice and Rats	4/20/2016 2:27:10 PM		900041344
Working with the IACUC	4/19/2016 2:31:15 PM		900041344
Working with Rats in Research Settings	4/15/2016 2:15:06 PM		900041344
Working with Mice in Research Settings	4/14/2016 11:13:46 AM		900041344
Working with Rabbits in Research Settings	4/14/2016 4:33:24 PM		900041344

ADMINISTRATIVE MANAGER

The Administrative Manager can view protocol and create animal orders. Click the Add button to add multiple Administrative Managers. Leave blank if you do not have an Administrative Manager.

Administrative Manager

Name	Affiliation	Will this person have direct contact with a live animal or assume direct responsibility for some aspect of the care and use of live animals under this protocol?
Trenace Richardson	Staff	N
Teri Herbert	Other	N

Administrative Manager

Name*
Trenace Richardson

Degree(s)

Email*
washitre@musc.edu

Phone*
8437928584

Title
Administrative Assistant

Affiliation*
Staff

Will this person have direct contact with a live animal or assume direct responsibility for some aspect of the care and use of live animals under this protocol?* N

Describe general experience/training related to animal research. Species- and procedure-specific experience will be addressed in the "Procedures" section of the application.

Protocol Title: Polo-like kinase 1 (Plk1) inhibition for the treatment of breast cancer

Training Details
No training data is available.

Administrative Manager

**Name***	**Degree(s)**
Teri Herbert	
**Email***	**Phone***
herbertl@musc.edu	843-792-1370
**Title**	**Affiliation***
Associate Professor	Other

Will this person have direct contact with a live animal or assume direct responsibility for some aspect of the care and use of live animals under this protocol?* N

Describe general experience/training related to animal research. Species- and procedure-specific experience will be addressed in the "Procedures" section of the application.

Training Details
No training data is available.

OTHER PERSONNEL

List all individuals performing the experimental manipulations or working with animals. Individuals must have completed the IACUC training program before beginning work on this project. Individuals listed as 'Other Personnel' can only view the protocol.

EMERGENCY CONTACT INFORMATION

Will the PI be the primary contact for all animal care issues on this protocol?* Y

List persons to be contacted in case of an emergency or clinical deterioration of animals outside of normal working hours. Please include phone numbers, cell numbers, and pager numbers for each person listed. You must respond to this item.

Click Next to advance to every section of the protocol. Every time you click Next, your changes will be saved. If the page does not load, right click with your mouse and select Refresh/Reload.

---

* * * Species * * *

If the page does not load, right click with your mouse (MAC users press control-click) and select Refresh/Reload.

Resources: IACUC Website | OAR Website

This is a mandatory section. You must add at least 1 species to continue. Separate procedural information must be included for each species to be used.

Species to be Used

1. **Species Common Name** Mouse - Mus

2.	**Strain(s)**	NOD-scid-IL2 receptor gamma null female mice (NSG)
3.	**Animal Sex**	Female
4.	**Age Range**	-
5.	**Weight Range**	15 - 25 gm(s)
6.	**Housing Facility (animals will remain for 24 hours)**	DLAR only

7. **USDA Pain Category (Choose all that will apply. Enter the total number of the species to be used in each Pain Category. If animals will be used in more than one category, enter the number in the higher category. EXAMPLE: If 150 mice will be used in Category C for ear punching and 50 of those mice will then be used in Category D for laparotomy, list 100 mice in Category C and 50 mice in Category D)**

	**Pain Category C**	
X	**Pain Category D**	120
	**Pain Category E**	

8. **Maximum proposed number for this species** 120

9. **Source of Animals (please specify the source of animals; choose all that apply)**

X	**commercial Vendor(s)**
	**Transfer from Other Institution(s)**
	**Wild-Caught**
	**Transfer within MUSC**

10. **How will individual animals be identified? If animals will not be individually identified, explain how groups will be identified.**

    Animals will be identified by ear-tagging with individually numbered small rodent ear tags (National Band and Tag Cat#1005-1). Tags will be placed in the right ear during initial procedure and under anesthesia to minimize distress.

11. **Have any of the animals undergone procedures prior to being used on this protocol?** N

    Please specify which animals underwent procedures, what procedures were performed, and where those procedures were performed.

Description of Phenotypes

12. **Do any of the strains listed for question 2 have a phenotype that could negatively impact animal health or animal care? This includes animals which are immunocompromised; spontaneously develop tumors or other disease states; exhibit tremors or other distinctive behaviors; or present with specific conditions like alopecia that might cause concern even if clinically irrelevant.** Y

    a. **Describe for each impacted strain any expected characteristic clinical signs or abnormal behavior related to the genotype.**

    NSG are immunocompromised. Should spontaneous tumors develop, or any unforseen complications, we will consult the veterinarian staff for the use of any medication.

    b. **Describe for each impacted strain any measures intended to minimize pain and distress, or customized husbandry required to manage potential problems.**

    If any animals display signs of pain or distress, the mice will be euthanized by CO2 inhalation and subsequent cervical dislocation.

13. **Explain why the species indicated is particularly appropriate to the work proposed (PHS Policy, IV.D.1.b) Consider such characteristics as body size, availability of specific strains, breeds, or mutants, data from previous studies, and unique anatomic or physiologic features. Explain why these are important to the work proposed.**

    We will use the NOD-scid-IL2 receptor gamma null female mice (NSG) to generate in vivo PDXs mice models. This is a well-characterized mouse strain able to generate PDX models. We will run a pilot PDX Clinical Trial (PCT), to test the efficacy of PLK-1i (Onvansertib) in reducing TNBC tumor growth when used as an individual agent or in conjunction with paclitaxel. We have selected a cohort of TNBC PDX models from respective individual patients representing the spectrum of TNBC genomic and transcriptional profiles available to us.

Special Considerations

You must respond to questions 1-9 with either Yes or No. If yes, you will be required to give additional information.

1. Will you be using one or more MUSC Core Facilities? * N

2. Will you be using any live animals for teaching or training? * N

Note: If protocol is solely for training, all trainers/key personnel must be listed as personnel on this protocol, but trainees do not need to be individually listed. No experimental aims may be included on a training protocol. Experimental protocols with a training component must list all personnel, including trainees, who will have contact with a live animal under this protocol. For all animals used for training purposes, any potentially painful or distressful activities must occur under anesthesia as part of a single terminal procedure.

a. What is the specific purpose of this use? (e.g., "Training is sole use of protocol", or "Subset of animals will be used to train protocol personnel on proposed experimental procedures")

b. What species and approximately how many animals will be used for teaching or training?

c. Explain what other training activities are used that don't involve live animals, and why they are not sufficient for the need.

d. List the procedures included in this protocol that will be conducted on the training animals. If sole purpose of protocol is for training, state "All procedures".

3. Biological Materials

a. Are you using microbial or infectious agents, biotoxins or recombinant DNA? * N

The use of microbial or infectious agents (e.g., lentiviral vectors, adenoviral vectors, etc.), biotoxins or rDNA in animals must be approved by the Institutional Biosafety Committee.

b. Are you using Cells, cell lines or tissues? * Y

Cells/cell lines or tissues

Species	Cell/cell line or tissue proposed for use in this species	Specify type of biological material	Is the material of rodent origin or has it been passaged through rodents?
Mouse - Mus	PDX tissue (TM00090, TM00099 and TM00999)	Patient-Derived Tissue	Y

Cells/cell lines or tissues

3.b.i Species * — Mouse - Mus

3.b.ii Cell/cell line or tissue proposed for use in this species* — PDX tissue (TM00090, TM00099 and TM00999)

3.b.iii Specify type of biological material * — Patient-Derived Tissue

	Describe source of material (e.g., ATCC, MUSC biorepository, or Dr. XY at ABC University)	JAX Laboratory
3.b.iv	Species of origin *	Human
3.b.v	Is the material of rodent origin or has it been passaged through rodents?	Y

[IF YES] Test results will be required. Upload results or contact dlardiag@musc.edu if you need to request testing services.

3.b.vi	Route, site, number, and frequency of injections or administrations *	mammary fat pad, once
3.b.vii	Dose and volume of injection or administration *	100 ul of PDX
3.b.viii	Expected length of survival time after last administration *	8 weeks or or until tumor volumes reached the 1,500-mm3 end point
3.b.ix	Modification with rDNA *	N
3.b.x	Modification source:	
3.b.xi	Host system of rDNA *	
3.b.xii	Describe rDNA insert.	
3.b.xiii	BSO notes.	

Once animals are implanted with human tissue, cages will be identified with a "Human cell hazard" sticker.

3.b.xiv	Names of personnel who will be using the substance *	Antonio Giordano, Elizabeth Yeh, Yueying Liu

c. Are you using Other Biological Materials? * N

4. Chemical Hazards

Are you using chemical agents carying more than minimal risk, including chemcial toxins, chemotherapy agents, carcinogens or potentially explosive materials in animals? * Y

Chemical Hazards

Species	Chemical Hazard
Mouse - Mus	Paclitaxel
Mouse - Mus	Onvansartib

Chemical Hazards

4.a.	Chemical Hazard *	Paclitaxel
4.b.	Species *	Mouse - Mus
4.c.	Route of Administration *	Intraperitoneal (IP)
4.d.	Dosage (in mg/kg if possible) and volume of injection or administrations *	10-30 mg/kg 1-7 times per week, for 2-4 weeks
4.e.	Site and number of injections or administrations *	one, peritoneum
4.f.	Expected length of survival time after last administration *	4 weeks or tumor < 3,375-mm3 end point

4.g. List handling instructions, required PPE, and length of time hazard will be present in cage environment after administration. Approved Chemical Hazards Appendix must be submitted as attachment.

Investigator will use aseptic technique including sterile gloves and autoclave sterilized tools. Sterile gloves, sterile gown, surgical cap and face mask will be used.

4.h. Names of personnel who will be using the substance * — Antonio Giordano, Elizabeth Yeh, Yueying Liu

Chemical Hazards

4.a. Chemical Hazard * — Other

Onvansartib

4.b. Species * — Mouse - Mus

4.c. Route of Administration * — Oral (PO)

4.d. Dosage (in mg/kg if possible) and volume of injection or administrations * — 60-120 mg/kg/day for 2 days, every week, for 2-4 weeks

4.e. Site and number of injections or administrations * — oral, day 1-2, q8

4.f. Expected length of survival time after last administration * — 4 weeks or tumor < 3,375-mm3 end point

4.g. List handling instructions, required PPE, and length of time hazard will be present in cage environment after administration. Approved Chemical Hazards Appendix must be submitted as attachment.

Investigator will use aseptic technique including sterile gloves and autoclave sterilized tools. Sterile gloves, sterile gown, surgical cap and face mask will be used.

4.h. Names of personnel who will be using the substance * — Antonio Giordano, Elizabeth Yeh, Yueying Liu

5. Radiological Agents

Are you administering radioactive agents to live animals? * — N

The use of radioactive substances must be approved by the Radiation Safety Committee.

6. Field Study or Wildlife Study* — N

7. Will any non-pharmaceutical-grade compounds be administered to live animals?* — N

8. Will any controlled substances be administered under this protocol?* — N

---

* * * Funding * * *

If the page does not load, right click with your mouse (MAC users press control-click) and select Refresh/Reload.

Funding

Except for Department of Defense grants, multiple grants may be used to support the proposed work. Prior to ordering animals on this protocol, it will be necessary to obtain UDAKs for each funding source, which will be entered in the animal ordering system. ORSP will initiate a congruency check between the grant and the supporting protocol(s) for all PHS-funded projects.

Conflict of interest *

Do any of the participating study investigators or other research personnel (or their immediate family) have a — N

financial and/or intellectual property interest in the sponsor or products used for this research study?

Department of Defense *

Will Department of Defense funds be used to support any part of the proposed project? N

Are there any Memoranda of Understanding or Interinstitutional Assurance documents associated with this protocol? If yes, the document must be uploaded as an attachment. N

Please note that if Department of Defense funding is listed, no other work should be described and no other funding sources may be included on this protocol.

---

* * * Rationale * * *

If the page does not load, right click with your mouse (MAC users press control-click) and select Refresh/Reload.

Resources: IACUC Website | OAR Website

MUSC Protocol ID (for office use only)

Official Project Title

Polo-like kinase 1 (Plk1) inhibition for the treatment of breast cancer

Items marked with red stars (*) are required by the system to construct the protocol.

Please respond to all applicable items in the form to facilitate the approval of your protocol by the IACUC. If an item does not apply, respond with N/A.

Note: Use language understandable to a layperson as you answer questions in this section. Avoid overly technical terms and define abbreviations.

You can check the language level by pasting your text into the online tool available at http://www.online-utility.org/ english/ readability_test_and_improve.jsp.

How to Complete These Sections: Provide as much detail as needed for this section. You may copy and paste plain text from a document into the text boxes (Please note: Formatting will not transfer into the text box). Please separate paragraphs with a blank line. If you have tables or diagrams, please use the Add feature to attach the document in the Attachments section.

Study Details

1. Aims and Significance

a. Lay summary. Using non-technical (lay) language that a senior high school student would understand, summarize the conceptual design of the experiment in no more than one or two paragraphs.*

Traditional chemotherapeutic regimens kill cells that are in the process of splitting into 2 new cells. Because cancer cells divide much more often than most normal cells, chemotherapy is much more likely to kill them. The fact that chemotherapy drugs kill dividing cells helps to explain why chemotherapy causes side effects. It affects healthy body tissues where the cells are constantly growing and dividing. Recently, research has identified cancer-specific targets that have led to the development of a new generation of targeted cancer therapeutics. These therapies have revolutionized how cancer is treated in the clinic today and have led to improved patient survival outcomes while at the same time significantly reducing negative side effects. We have found that polo-like kinase 1 (PLK1) inhibitors synergize with conventional chemotherapy, such as taxane, in breast cancer cell lines. It is now critical for us to test this approach in an in vivo tumor growth model. If successful, this data will serve as the next step in our development of this strategy for use in clinical trials for the treatment of breast cancer patients, which is a disease that claims the lives of >40,000 women annually in the U.S. alone.

b. Description of relevance and harm/benefit analysis:

Using non-technical (lay) language that a senior high school student would understand, briefly describe how this research project is intended to improve the health of people and/or other animals, or otherwise to serve the good of society, and explain how these benefits outweigh the pain or distress that may be caused in the animals that are to be used for this protocol.*

For these studies, we plan to implant human breast cancer cells into the mammary gland of mice and allow these cells to form a tumor. Mice will then be treated with drugs to induce cancer shrinkage. Tumor size will be monitored to determine if drugs

tumor. Mice will then be treated with drugs to induce cancer shrinkage. Tumor size will be monitored to determine if drugs effectively shrink the tumors in the mice.

2. Groups and Group Sizes

a. Summarize the design of the experiment in terms of the specific groups of animals to be studied. Group sizes are addressed below. This summary should be an overview of experimental groups included in the project.*

For these studies, we plan to implant human breast cancer cells into the mammary gland of mice and allow these cells to form a tumor. Mice will then be treated with a combination of targeted drugs that have been rationally designed based on the genetic characteristics of the cell line in order to induce synergistic cancer cell death. Tumor growth will be monitored to determine if the rationally designed therapeutic combinations effectively result in breast cancer cell death and tumor shrinkage in vivo.

We plan to have several groups of mice for this study. All groups will have human breast cancer cells (patient derived xenograft) implanted into the mammary fat pad. Once tumors reach a size of 100 cubic millimeters groups of mice will be treated with one the following drugs or drug combinations:
- Placebo
-Paclitaxel
-Onvansertib
-Paclitaxel + Onvansertib

4 groups X 2 PDX modelx X n=10 = 80; we think we may need ro repeat about half of the trials for confirmation of results = 80 + 40 = 120.

b. Justify the group sizes and the total number of animals requested, including all control groups. A power analysis is strongly encouraged. A table may be attached in addition to the written justification.*

The study will also include a three-animal vehicle arm to control for standard tumor growth. We estimate that a group sample size of eight will result in power of 95% to discern a difference of 50% growth between any treatment arm within a PDX model. This sample size will provide power of 90% to discern a difference of 25% growth between arms.

* * * Procedures * * *

Study of effects of drugs or toxins in vivo

Resources: IACUC Website | OAR Website

1.	**Procedure Type:**	Study of effects of drugs or toxins in vivo
2.	**Brief Description:**	We will run a pilot PDX Clinical Trial (PCT), to test the efficacy of PLK-1 inhibitor (Onvansertib) in reducing TNBC tumor growth when used as an individual agent or in conjunction with paclitaxel
3.	**Species:**	Mouse - Mus
4.	**USDA Pain/Distress Category:**	D
5.	**Approximate number of animals to be used in this procedure at this location:**	120

Protocol Title: Polo-like kinase 1 (Plk1) inhibition for the treatment of breast cancer

* * * Procedure Description * * *

Procedure Description

1. Detailed Procedure Description

Oral gavage will be performed for the delivery of the targeted drugs as follows: the PLK1 inhibitor Onvansertib (Trovagene Oncology, San Diego, CA, USA) will be administered by oral gavage per os over 2 days with a 5 day rest for 2-4 weeks. In this procedure a stainless steel bulb tipped gavage needle or a flexible cannula or tube is attached to a syringe and used to deliver the compound into the stomach. The recommended maximum volume for administration is 1% of body weight (e.g., a 20 gm mouse can be given 0.2 ml). The animal will be gently restrained (grasping the animal by the loose skin of the neck and back) to immobilize the head but not such that the animal vocalizes or shows other signs of distress. Maintaining the animal in an upright (vertical) position, we will pass the gavage needle along the side of the mouth. After the needle is passed to the correct length, the compound will be injected. Flexible plastic gavage needles will be used to limit potential esophageal distress to the animal.

Paclitaxel will be administered by i.p. injection (J Natl Cancer Inst. 1996 Sep 18;88(18):1308-14. PMID: 8797771). The injection of substances directly into the peritoneum will be done in strict asepsis. Injected substances and the needles/syringes used to inject substances must be sterile as described. Animals will received treatments 1-7 time/week. This procedure is expected to only cause momentary distress.

Duration of treatment will be between 2 weeks to 4 weeks depending on how tumors respond.

2. Please list and describe any clinical effects or changes from the normal health and behavior of an untreated animal which may occur as a result of this procedure.

All experiments described in this protocol include mammary tumor production as a study endpoint. Although primary mammary tumors should pose no potential for distress, other than possible ulceration, metastasis into other organ systems is possible and may result in rapid decline in health. Any animal exhibiting signs of respiratory distress, moribundity, extreme lethargy, inability to ambulate normally, or have a body condition score < 2/5 will be euthanized immediately. Animals that are hunched and scruffy in appearance, exhibiting decreased levels of activity but alert and active when manipulated, or with a body condition score of < 3/5 will be supported with diet supplementation on the cage floor and the vet staff contacted for subcutaneous fluids. In the case of tumor ulceration, the veterinarian will be notified immediately and asked to perform clinical evaluation to determine if the condition warrants treatment or immediate euthanasia.

3. Describe post procedure monitoring, observation schedules, and treatment that will be performed.

Health monitoring and tumor measurements will be performed by laboratory personnel listed on this protocol, weekly. Body weights will be continuously monitored throughout each experiment. Treatment options or euthanasia for cases of tumor ulceration will be determined under the guidance of the veterinarian. Possible treatments may include daily application of topical anesthetic and/or triple antibiotic ointment to the affected area. Mice undergoing wound treatment will be evaluated daily for indications of treatment success.

Ulceration is justified and addressed as following:
Ulceration of a tumor will not by itself criteria for sacrificing an animal. Successful immune-based therapies can often cure mice that have tumor ulceration, and ulcerating tumors can completely regress. Successful therapy may itself lead to transient ulceration of the tumor as the immune cells are destroying the tumor from within. Transient ulceration is not expected to lead to additional pain, and in fact, may be accompanied by tumor regression, and improved outcome and survival. To ensure that pain and discomfort is minimized, mice with ulcerating tumors will be monitored as described above and euthanized upon recommendation of DLAR and the veterinary staff.

Protocol Title: Polo-like kinase 1 (Plk1) inhibition for the treatment of breast cancer

We will also use the following criteria for euthanasia if ulceration does not resolve. Thus, mice with any of the following criteria will be sacrificed:

1) Ulcers do not heal or form scabs within 7 days
2) If tumor area increases by 30% (and the initial tumor measurement is at least by 100mm2)
3) If mouse shows sign of dehydration, lethargy, etc.

4. Are expected or potential effects from this specific procedure likely to result in more than momentary or slight pain or distress to the animals? Y

4a. Will analgesics be administered to minimize pain and distress? N

Provide justification for withholding analgesics.

IP injections and oral gavage can be done in conscious mice without causing distress

4b. Describe the criteria specific to this procedure which will be used to determine when an animal should be euthanized or referred for clinical treatment.

Although primary mammary tumors should pose no potential for distress, unexpected events include possible ulceration and metastasis into other organ systems, which may result in rapid decline in health. Any animal exhibiting signs of distress such as ulceration, poor body condition, lethargy, piloerection, and lack of grooming behavior will be euthanized for tissue harvest.

Protocol Title: Polo-like kinase 1 (Plk1) inhibition for the treatment of breast cancer

* * * Personnel & Location * * *

Personnel Details - provide requested information for each person who will perform or directly participate in this procedure.

Location of Work– provide information on any non-DLAR locations where this specific procedure will be performed. List one non-DLAR location per entry.

Personnel Details

Personnel Name	Specific Procedure Experience
Antonio Giordano	Y
Yueying Liu	Y
Elizabeth Yeh	Y

Personnel Details

1. **Name of person performing procedure** — **Antonio Giordano**
2. **Does this person have prior experience with this procedure on this species?** — Y
3. **Describe the previous experience and/or training plan to ensure proficiency with this procedure with this species.**

   Training for Antonio Giordano will be provided by Elizabeth Yeh who have extensive experience with the procedures. Dr. Giordano has direct experinece of animal, both mouse and rat, blood collection (tail) as graduate student.

1. **Name of person performing procedure** — **Yueying Liu**
2. **Does this person have prior experience with this procedure on this species?** — Y
3. **Describe the previous experience and/or training plan to ensure proficiency with this procedure with this species.**

   Additional training for Yueying Liu will be provided by Elizabeth Yeh who have extensive experience with the procedures.

1. **Name of person performing procedure** — **Elizabeth Yeh**
2. **Does this person have prior experience with this procedure on this species?** — Y
3. **Describe the previous experience and/or training plan to ensure proficiency with this procedure with this species.**

   The investigator has >15 years experience in performing xenograft tumor assays

---

Protocol Title: Polo-like kinase 1 (Plk1) inhibition for the treatment of breast cancer

* * * Anesthesia & Analgesia * * *

1. Will anesthesia be administered for this procedure? N

Anesthetic Regimen

1a. Parameters used to monitor and ensure appropriate anesthetic depth.

1b. Anesthetic Agents

1c. Paralytic Agents (may only be used with anesthesia)

1d. Will animals be recovered after anesthesia?

1e. Describe how animals will be monitored for recovery from anesthesia. Address parameters to be monitored, schedule and frequency of observations.

Immediate post-procedure period ends when animals are awake and ambulatory.

2. Will analgesia be administered for this procedure? N

2a. Analgesic Regimen

Protocol Title: Polo-like kinase 1 (Plk1) inhibition for the treatment of breast cancer

* * * Other Drugs Utilized * * *

Other drugs utilized for this procedure

Other Drugs Utilized

Agent Name	Purpose of Drug
Paclitaxel	decrease tumor size
Onvansertib	decrease tumor size

Other Drugs Utilized

1. **Agent Name** — **Paclitaxel**
2. **Dosage (in mg/kg if possible) AND Volume of Administration (when applicable)** — 10-30 mg/kg 100 -200 ul
3. **Route** — **Intraperitoneal (IP)**
4. **Purpose of Drug**
   decrease tumor size
5. **Duration and Frequency of Administration**
   1-7 times per week, 2-4 weeks

1. **Agent Name** — **Other**
   Onvansertib
2. **Dosage (in mg/kg if possible) AND Volume of Administration (when applicable)** — 60-120 mg/kg 100-200uls
3. **Route** — **Gavage**
4. **Purpose of Drug**
   decrease tumor size
5. **Duration and Frequency of Administration**
   2 times per week, 2-4 weeks

Experimental neoplasia

Resources: IACUC Website | OAR Website

Protocol Title: Polo-like kinase 1 (Plk1) inhibition for the treatment of breast cancer

1.	**Procedure Type:**	Experimental neoplasia
2.	**Brief Description:**	PDX injection
3.	**Species:**	Mouse - Mus
4.	**USDA Pain/Distress Category:**	D
5.	**Approximate number of animals to be used in this procedure at this location:**	120

---

* * * Procedure Description * * *

Procedure Description

1. **Detailed Procedure Description**

For tumor cell injections, animals may be anesthetized followed by injection of a solution containing patient derived xenograft models TM00090-099-999 (JAX Laboratory). Tumor specimens are isolated and minced into small (1x1x1) mm<3>-(5x5x5) mm<3> fragments that are then implanted subcutaneously (SC) into the right hind flanks of 5-7 wk old NSG mice. Heating pad will be provided for the animal to help regulate body temperature if anesthesized. Animals will be monitored during the procedure for signs of distress (change in body temperature, rate of breathing, movement or vocalization associated with pain). If signs of distress are observed, increased time or level of exposure of isofluorane will be used to ensure the animal is sedated.

2. **Please list and describe any clinical effects or changes from the normal health and behavior of an untreated animal which may occur as a result of this procedure.**

All experiments described in this protocol include mammary tumor production as a study endpoint. Although primary mammary tumors should pose no potential for distress, other than possible ulceration, metastasis into other organ systems is possible and may result in rapid decline in health.

3. **Describe post procedure monitoring, observation schedules, and treatment that will be performed.**

Health monitoring and tumor measurements will be performed by laboratory personnel listed on this protocol, weekly. Body weights will be continuously monitored throughout each experiment. Treatment options or euthanasia for cases of tumor ulceration will be determined under the guidance of the veterinarian.

Ulceration of a tumor will not by itself criteria for sacrificing an animal. Successful immune-based therapies can often cure mice that have tumor ulceration, and ulcerating tumors can completely regress. Successful therapy may itself lead to transient ulceration of the tumor as the immune cells are destroying the tumor from within. Transient ulceration is not expected to lead to additional pain, and in fact, may be accompanied by tumor regression, and improved outcome and survival. To ensure that pain and discomfort is minimized, mice with ulcerating tumors will be monitored as described above and euthanized upon recommendation of DLAR and the veterinary staff.

We will also use the following criteria for euthanasia if ulceration does not resolve. Thus, mice with any of the following criteria will be sacrificed:

1) Ulcers do not heal or form scabs within 7 days

1) Ulcers do not heal or form scabs within 7 days
2) If tumor area increases by 30% (and the initial tumor measurement is at least by 100mm2)
3) if mouse shows sign of dehydration, lethargy, etc.

4. Are expected or potential effects from this specific procedure likely to result in more than momentary or slight pain or distress to the animals? Y

4a. Will analgesics be administered to minimize pain and distress? Y

Provide justification for withholding analgesics.

4b. Describe the criteria specific to this procedure which will be used to determine when an animal should be euthanized or referred for clinical treatment.

Although primary mammary tumors should pose no potential for distress, unexpected events include possible ulceration and metastasis into other organ systems, which may result in rapid decline in health. Any animal exhibiting signs of distress such as ulceration, poor body condition, lethargy, piloerection, and lack of grooming behavior will be euthanized for tissue harvest. Ulceration is justified in Section 3.

Protocol Title: Polo-like kinase 1 (Plk1) inhibition for the treatment of breast cancer

* * * Personnel & Location * * *

Personnel Details - provide requested information for each person who will perform or directly participate in this procedure.

Location of Work– provide information on any non-DLAR locations where this specific procedure will be performed. List one non-DLAR location per entry.

Personnel Details

Personnel Name	Specific Procedure Experience
Antonio Giordano	Y
Yueying Liu	Y
Elizabeth Yeh	Y

Personnel Details

1. **Name of person performing procedure** — **Antonio Giordano**
2. **Does this person have prior experience with this procedure on this species?** — Y
3. **Describe the previous experience and/or training plan to ensure proficiency with this procedure with this species.**

   Training for Antonio Giordano will be provided by Elizabeth Yeh who have extensive experience with the procedures. Dr. Giordano has direct experinece of animal, both mouse and rat, blood collection (tail) as graduate student.

1. **Name of person performing procedure** — **Yueying Liu**
2. **Does this person have prior experience with this procedure on this species?** — Y
3. **Describe the previous experience and/or training plan to ensure proficiency with this procedure with this species.**

   Additional training for Yueying Liu will be provided by Elizabeth Yeh who have extensive experience with the procedures.

1. **Name of person performing procedure** — **Elizabeth Yeh**
2. **Does this person have prior experience with this procedure on this species?** — Y
3. **Describe the previous experience and/or training plan to ensure proficiency with this procedure with this species.**

   The investigator has >15 years experience in performing xenograft tumor assays

---

Protocol Title: Polo-like kinase 1 (Plk1) inhibition for the treatment of breast cancer

* * * Anesthesia & Analgesia * * *

1. Will anesthesia be administered for this procedure? Y

Anesthetic Regimen

1a. Parameters used to monitor and ensure appropriate anesthetic depth.

paw pinch, monitor breathing, body temperature, movement, and vocalization by the animal

1b. Anesthetic Agents

Anesthetic Agents

Agent Name	Route	Dosage (in mg/kg if possible) AND Volume of Administration (when applicable)	Duration and Frequency of Administration
Isoflurane	Inhalation (IN)	2.5-4% mixed with O2 or air	1-5 minutes

Anesthetic Agents

1. Agent Name — Isoflurane
2. Dosage (in mg/kg if possible) AND Volume of Administration (when applicable) — 2.5-4% mixed with O2 or air
3. Route — Inhalation (IN)
4. Duration and Frequency of Administration. If using an inhalation agent, describe how it is applied.

   1-5 minutes

1c. Paralytic Agents (may only be used with anesthesia)

1d. Will animals be recovered after anesthesia? Y

1e. Describe how animals will be monitored for recovery from anesthesia. Address parameters to be monitored, schedule and frequency of observations.

Immediate post-procedure period ends when animals are awake and ambulatory.

paw pinch, monitor breathing, body temperature, movement, and vocalization by the animal

2. Will analgesia be administered for this procedure? N

2a. Analgesic Regimen

Protocol Title: Polo-like kinase 1 (Plk1) inhibition for the treatment of breast cancer

* * * Other Drugs Utilized * * *

Other drugs utilized for this procedure

Other Drugs Utilized

Agent Name	Purpose of Drug
TM00090,099 and 999 (JAX Laboratory)	to cause cancer

Other Drugs Utilized

1. **Agent Name** — **Other**
   TM00090,099 and 999 (JAX Laboratory)
2. **Dosage (in mg/kg if possible) AND Volume of Administration (when applicable)** — tumor fragment of (1x1x1)mm3-(5x5x5)mm3
3. **Route** — **Subcutaneous (SC)**
4. **Purpose of Drug**
   to cause cancer
5. **Duration and Frequency of Administration**
   once

---

* * * Alternative Search * * *

If the page does not load, right click with your mouse (MAC users press control-click) and select Refresh/Reload.

The 3 Rs - Replacement, Reduction and Refinement - are an integral component of conducting humane, ethical research. These are important to minimizing potential harm and maximizing benefit to be derived from the work.

Library resources, including direct searches performed by a reference librarian, are available without cost to MUSC faculty to assist with completion of this section.

Resources: IACUC Website | OAR Website

1. Literature Search for Alternatives.

Search Data

Name of Potentially Painful or Distressful Procedure	Search Range From	Search Range To
Induction of breast cancer, chemotherapy/PLK1 administration	2010	2019

Search Data

Protocol Title: Polo-like kinase 1 (Plk1) inhibition for the treatment of breast cancer

1.a.	Name of Potentially Painful or Distressful Procedure*	Induction of breast cancer, chemotherapy/PLK1 administration	
1.b.	Search Range From*	2010	(YYYY)
1.c.	Search Range To*	2019	(YYYY)
1.d.	Search Date*	01/18/2019	(MM/DD/YYYY)

Note: Because this is a search for alternatives to painful or distressful procedures, you are advised to use the word "alternative" as a search term along with words that describe the painful procedures described in this protocol.

1.e.	Keywords*	PLK1/polo-like kinase 1; cancer/neoplasms/carcinoma; paclitaxel/onvansartib AND model OR cadaver OR abattoir OR cultured OR "animal use alternative" OR "in vitro" OR "non animal" OR IRAG OR "minimize animal" OR alternatives

1.f. Databases Searched*

X	Agricola Database		Alternatives to Animal Use in Research, Testing and Education
	Animal Welfare Info Center		ATLA (Alternatives to Laboratory Animal Journal)
	Benchmarks		BioOne
	BIOSIS		CAB Abstracts
	Current Contents	X	CRISP
	Google Scholar	X	Lab Animal
X	Lab. Animals Journal	X	Lab. Animal Sci. Journal
	Lab Animal Welfare Bibliography (QL55L27311988)	X	MEDLINE
X	Pubmed		PrimateLit
	Public STINET		Quick Biblio. Series
	REE	X	SCOPUS
	TOXLINE		TOXNET
	Web of Science	X	Other
			SciFinder, NIH RePORTer, ALTBIB, CRIS, NORINA, Zebet.

2. Replacement. Describe the replacements that have been incorporated into this work, the replacements that have been considered but cannot be used, and the reason(s) that further replacements are not acceptable. (e.g., computer modeling, in vitro cell or tissue cultures, insect models)

We have made extensive use of in vitro cultured cell line experiments in order to identify the synergistic drug combination being examined in this study. Many potential combinations have been ruled out through our in vitro work and will therefore not be pursued in vivo. The drug combinations being examined in this protocol has demonstrated striking in vitro effectiveness, the next logical step now is to examine it in an in vivo mammalian model system. Any novel therapeutic approach that is effective at killing cancer cells in vitro must be tested in in vivo mammalian models that more closely mimic the environment of tumor cells growing in situ in human patients.

3. Reduction. Describe how the number of animals to be used has been minimized in this protocol and explain why further reduction would disproportionately compromise the value of the data. (e.g., use multiple samples from a single animal for different aims, use published data on control/group sizes)

The number of animals required for this study has been carefully considered and we are proposing to use the minimum number of animals required to achieve statistical significance while taking into consideration inherent variability associated with disease modeling. These calculations were based on published historical data where we performed similar types of experiments. However, the experiments we propose are novel and will not duplicate these results. It is important to point out that all experiments are proposed to test a defined hypothesis and by definition, these hypotheses could be incorrect. In cases where our initial pilot experiments demonstrate our hypothesis is incorrect, we may not pursue further investigation and use less numbers of mice than originally proposed.

4. Refinement. Describe the refinements that have been incorporated into this work and explain why no further refinements are feasible. (e.g., less-invasive procedures, techniques that minimize pain or distress)

The current protocol has inherent procedures to limit pain and discomfort for the animals. We aim to perform procedures in the most

minimally invasive manner. Injections of cells and pharmacological agents are per local skin route or delivered orally to minimize pain. Refinements for these procedures include use of fine gauge needles for tumor cell delivery and use of flexible gavage needles for minimum discomfort during this procedure, and use of isofluorane as an anesthetic which generally provides easy and quick recovery for the mice. In all experiments, when any potential adverse health issues are raised, mice will be carefully monitored according to the distress guidelines outlined in the "Guide for the Care and Use of Laboratory Animals." However, any animals exhibiting signs of severe illness (e.g. weight loss, extreme loss of motility, extremely moribund state, open wounds etc.) will be humanely euthanized immediately. Animals, food, and water will be inspected daily and cages, including bedding will be changed periodically.

5. Describe how it was determined that the proposed work does not unnecessarily duplicate work already documented in the literature.

A search was done to determine that this research is not unnecessarily duplicative and alternatives are not an option.

6. Alternatives for Category E Procedures

For Category E procedures, explain why pain relieving drugs or other ameliorative treatments cannot be used to alleviate pain/distress.

----

* * * Timing & Endpoints * * *

If the page does not load, right click with your mouse (MAC users press control-click) and select Refresh/Reload.

Resources: IACUC Website | OAR Website

Timing & Endpoints

1. **Please describe the sequence and timing of all the manipulations for each group of animals. Also include the time between procedures. Use enough detail to allow reviewers to understand what each animal may undergo. Please separate paragraphs with a blank line**

Breast cancer cell injection ->

Weekly measurement of tumor growth by electronic calipers ->

Once tumors reach 5 mm diameter, drug treatment 1-2 times per week will be initiated ->

Continued weekly measurement of tumor growth by electronic calipers ->

Euthanization for tissue collection (up to 4 months post-injection)

Note: You may copy and paste plain text from a document into the text boxes (Formatting will not transfer into the text box).

Recommendation: When copying text from a Word or PDF file, it is recommended to first paste into Notepad, and then copy and paste into the eProtocol text box.

If you have tables or diagrams that will assist with the understanding of the experimental animal groupings, please use the Add feature to attach the document in the Attachments section of Protocol Information. Reference the name of the attachment in the text of the description where appropriate.

2. **Flow Chart - If the protocol involves more than one procedure (i.e., simple euthanasia and tissue harvest), please submit a procedures flow chart with this protocol. The flow chart should illustrate/include in chronological order all the procedures that the animals will undergo starting with their arrival on the protocol and ending with their euthanasia or removal from the study. Indicate the timeline for the events (i.e., if animals are involved in multiple procedures, note the time period between procedures). Please use the Add feature to attach the document in the Attachments section of Protocol Information.**

Adverse Events

1. **Describe any common or expected consequences or complications that may arise and what will be done to address them.**

All experiments described in this protocol include mammary tumor production. In the case of minor tumor ulceration, the veterinarian will be notified immediately and asked to perform clinical evaluation to determine if the condition warrants treatment or immediate euthanasia. Ulceration justification is addressed in Procedure Description Section.

Health monitoring and tumor measurements will be performed by laboratory personnel listed on this protocol, weekly. Body weights will be continuously monitored throughout each experiment. Treatment options or euthanasia for cases of tumor ulceration will be determined under the guidance of the veterinarian. Possible treatments may include daily application of topical anesthetic and/or

triple antibiotic ointment to the affected area. Mice undergoing wound treatment will be evaluated daily for indications of treatment success.
If any animals display signs of pain or distress, the mice will be euthanized by CO2 inhalation and subsequent cervical dislocation.

2. **Specify anticipated morbidity and/or mortality rates associated with the procedures in this protocol. Complications in excess of anticipated morbidity rates or unexpected adverse events should result in a veterinary consult. Mortality rates more than 10% higher than the anticipated rate must be reported to the IACUC**

Although primary mammary tumors should pose no potential for distress, unexpected events include possible ulceration and metastasis into other organ systems, which may result in rapid decline in health. Any animal exhibiting signs of distress such as ulceration, poor body condition, lethargy, piloerection, and lack of grooming behavior will be euthanized for tissue harvest. The unexpected mortality is expected to be <10%.

Endpoint Criteria

1. **Experimental endpoints. Describe the criteria that will determine when animals will be removed from the protocol or euthanatized because they have reached the intended outcome of the experiment(s). If different groups have different intended outcomes, list each group separately.**

The defined experimental endpoint is when a mouse harbors a mammary gland tumor of 3.5 cm3 in diameter. Mice that do not develop tumors will be euthanized when they have surpassed twice the average tumor latency of the control group.

2. **Humane endpoints. Describe the protocol-specific criteria that will be used to determine when animals will be removed from the protocol or euthanatized prior to experimental endpoints to prevent suffering.**

Any animal exhibiting signs of respiratory distress, moribundity, extreme lethargy, inability to ambulate normally, or have a body condition score of < 2/5 will be euthanized immediately. Animals that are hunched and scruffy in appearance, exhibiting decreased levels of activity but alert and active when manipulated, or with a body condition score of 3/5 will be supported with diet supplementation on the cage floor and the vet staff contacted for subcutaneous fluids. In the case of tumor ulceration, the veterinarian will be notified immediately and asked to perform clinical evaluation to determine if the condition warrants treatment or immediate euthanasia.Any animal exhibiting signs of distress such as poor body condition, lethargy, piloerection, and lack of grooming behavior will be euthanized immediately. In the case of tumor ulceration, this condition warrants euthanasia and will be treated as a humane endpoint. Euthanasia for cases of tumor ulceration will be determined under the guidance of the veterinarian as described in Procedure Description Section. Health monitoring and tumor measurements will be performed by laboratory personnel listed on this protocol, weekly. Body weights will be continuously monitored throughout each experiment.

---

* * * Husbandry * * *

If the page does not load, right click with your mouse (MAC users press control-click) and select Refresh/Reload.
Resources: IACUC Website | OAR Website

1. Food or Fluid Regulation

Will food or fluid intake be regulated at any point during the proposed experimental procedures? This includes any scheduling or restriction of access time or volume. This DOES NOT include fasting immediately prior to surgery. N

2. Prolonged Conscious Restraint

Will physical restraint be used on conscious animals for more than momentary or brief periods during the proposed experimental procedures? N

3. Non-Standard Housing Environment

Are there any special requirements relating to cage/pen size, cage sanitation intervals, use of wire-bottom or disposable cages, or altered light cycles (i.e., not 12:12 diurnal schedule)? N

4. Non-Standard Social Environment

Will environmental enrichment be withheld, custom enrichment be used, or non-pregnant animals be deliberately single-housed for some or all of the time? N

5. Other Special Husbandry or Care

Are there any other special or unusual requirements for the care of animal subjects (e.g., special diet or supplements, special water, vibration-free)? N

---

* * * Disposition of Animals * * *

Protocol Title: Polo-like kinase 1 (Plk1) inhibition for the treatment of breast cancer

If the page does not load, right click with your mouse and select Refresh/Reload.

Resources: IACUC Website | OAR Website

Disposition of Animals

Species	Method of Disposition Primary
Mouse - Mus	CO2 from compressed gas tank
Mouse - Mus	Anesthetic overdose or veterinary euthanasia solution

Disposition of Animals

Species* — Mouse - Mus
Method of Disposition * Primary — CO2 from compressed gas tank
Gas exposure time — 5 minute
Method of Euthanasia (Secondary) — Cervical Dislocation
Will Euthanasia be performed within DLAR? — Y
Will Euthanasia be performed in PI lab(s)? — N
If yes, Specify Building/Room
Personnel Performing Procedure — Antonio Giordano, Elizabeth Yeh, Yueying Liu

Disposition of Animals

Species* — Mouse - Mus
Method of Disposition * Primary — Anesthetic overdose or veterinary euthanasia solution
Describe Euthanasia Method — isoflurane by inhaletion
Agent Name — Isoflurane
Route of Administration — Inhalation (IN)
Dosage (in mg/kg if possible) or if inhalation or immersion agent, the concentration — 5%
Method for verifying death. — Cervical dislocation
Will Euthanasia be performed within DLAR? — N
Will Euthanasia be performed in PI lab(s)? — Y
If yes, Specify Building/Room — DDB423
Personnel Performing Procedure — Antonio Giordano, Elizabeth Yeh, Yueying Liu

---

* * * Attachments * * *

If the page does not load, right click with your mouse (MAC users press control-click) and select Refresh/Reload.

Resources: IACUC Website | OAR Website

Please attach documents that provide direct support for this protocol.

Protocol Title: Polo-like kinase 1 (Plk1) inhibition for the treatment of breast cancer

If you have tables, diagrams, or other support documents, please use the Add button to attach the document(s).

Please name all attachments and reference those names in the appropriate narrative sections of the protocol.

Acceptable Attachment formats are: MS Word, MS Excel, MS PowerPoint, MS Visio, PDF, GIF, TIF, JPEG.

To update or revise any attachments, first delete the existing attachment and then add the revised document to replace it.

Document Type	Document Name	Attached Date	Submitted Date
Biological Materials Appendix	paclitaxel_MSDS	12/21/2018	01/11/2019
Biological Materials Appendix	CofA	12/21/2018	01/11/2019
Biological Materials Appendix	MSDS_NMS-1286937H	12/21/2018	01/11/2019
Chemical Hazard Approval	Giordano_00674	02/07/2019	02/07/2019
Chemical Hazard Approval	Giordano_00674_sign	02/07/2019	02/07/2019
Other	RB22 Health Report_Feb 2019	02/25/2019	02/25/2019

---

* * * Guidelines * * *

Mandatory (view and check Yes)

Use of Non-Pharmaceutical-Grade Chemicals and Other Substances — AGREE

Animal Acclimation — AGREE

Use of CO2 for Euthanasia of Rodents — AGREE

Non-Mandatory (view those relevant and check Yes)

Testing of Cell Lines and Biological Material — AGREE

Use of Toxic Substances

Unanticipated Phenotypes

Rodent Breeding Cage Density and Weaning

Aseptic Surgical Technique

Toe Clipping

Animals Used for Training Purposes
